# Recognition of Cell Wall Mannosylated Components as a Conserved Feature for Fungal Entrance, Adaptation and Survival Within Trophozoites of *Acanthamoeba castellanii* and Murine Macrophages

**DOI:** 10.3389/fcimb.2022.858979

**Published:** 2022-05-31

**Authors:** Marina da Silva Ferreira, Susana Ruiz Mendoza, Diego de Souza Gonçalves, Claudia Rodríguez-de la Noval, Leandro Honorato, Leonardo Nimrichter, Luís Felipe Costa Ramos, Fábio C. S. Nogueira, Gilberto B. Domont, José Mauro Peralta, Allan J. Guimarães

**Affiliations:** ^1^ Laboratório de Bioquímica e Imunologia das Micoses, Departamento de Microbiologia e Parasitologia, Instituto Biomédico, Universidade Federal Fluminense, Niterói, Brazil; ^2^ Pós-Graduação em Imunologia e Inflamação, Universidade Federal do Rio de Janeiro, Rio de Janeiro, Brazil; ^3^ Pós-Graduação em Doenças Infecciosas e Parasitárias, Faculdade de Medicina, Universidade Federal do Rio de Janeiro, Rio de Janeiro, Brazil; ^4^ Programa de Pós-Graduação em Ciências (Microbiologia), Instituto de Microbiologia Professor Paulo de Góes, Universidade Federal do Rio de Janeiro, Rio de Janeiro, Brazil; ^5^ Laboratório de Glicobiologia de Eucariotos, Instituto de Microbiologia Professor Paulo de Góes, Universidade Federal do Rio de Janeiro, Rio de Janeiro, Brazil; ^6^ Rede Micologia RJ - FAPERJ, Rio de Janeiro, Brazil; ^7^ Laboratório de Química de Proteínas, Departamento de Bioquímica, Instituto de Química, Universidade Federal do Rio de Janeiro, Rio de Janeiro, Brazil; ^8^ Departamento de Imunologia, Instituto de Microbiologia Paulo de Góes, Universidade Federal do Rio de Janeiro, Rio de Janeiro, Brazil; ^9^ Programa de Pós-Graduação em Microbiologia e Parasitologia Aplicadas, Instituto Biomédico, Universidade Federal Fluminense, Niterói, Brazil

**Keywords:** *Acanthamoeba*, macrophages, interaction, pathogenic fungi, mannose receptor

## Abstract

*Acanthamoeba castellanii* (*Ac*) is a species of free-living amoebae (FLAs) that has been widely applied as a model for the study of host-parasite interactions and characterization of environmental symbionts. The sharing of niches between *Ac* and potential pathogens, such as fungi, favors associations between these organisms. Through predatory behavior, *Ac* enhances fungal survival, dissemination, and virulence in their intracellular milieu, training these pathogens and granting subsequent success in events of infections to more evolved hosts. In recent studies, our group characterized the amoeboid mannose binding proteins (MBPs) as one of the main fungal recognition pathways. Similarly, mannose-binding lectins play a key role in activating antifungal responses by immune cells. Even in the face of similarities, the distinct impacts and degrees of affinity of fungal recognition for mannose receptors in amoeboid and animal hosts are poorly understood. In this work, we have identified high-affinity ligands for mannosylated fungal cell wall residues expressed on the surface of amoebas and macrophages and determined the relative importance of these pathways in the antifungal responses comparing both phagocytic models. Mannose-purified surface proteins (MPPs) from both phagocytes showed binding to isolated mannose/mannans and mannosylated fungal cell wall targets. Although macrophage MPPs had more intense binding when compared to the amoeba receptors, the inhibition of this pathway affects fungal internalization and survival in both phagocytes. Mass spectrometry identified several MPPs in both models, and in silico alignment showed highly conserved regions between spotted amoeboid receptors (MBP and MBP1) and immune receptors (Mrc1 and Mrc2) and potential molecular mimicry, pointing to a possible convergent evolution of pathogen recognition mechanisms.

## Introduction

Free-living amoeba (FLAs) are single-cell ubiquitous protozoa, found in environments such as soil, air, fresh and saltwater, dust, as well as men-built devices such as air conditioning units and cooling towers (Fonseca et al., 2020; Lee et al., 2020; Paknejad et al., 2020; Wopereis et al., 2020). The ubiquitous conditions of some FLAs, including *Acanthamoeba* spp., facilitate their access to susceptible hosts, making *Acanthamoeba* an opportunistic pathogen to humans and other mammals (Marciano-Cabral and Cabral, 2003; Schuster and Visvesvara, 2004; Visvesvara et al., 2007; Ghadage et al., 2017; Liu et al., 2019). In fact, studies show that more than 80% of individuals have circulating antibodies against *Acanthamoeba* antigens (Chappell et al., 2001). *Acanthamoeba* sp. can cause keratitis (AK), by direct exposure of the eyes to contaminated water or lack of hygiene when wearing contact lenses, posing serious risk of blindness if left untreated (Lorenzo-Morales et al., 2013; Carnt and Stapleton, 2016; Neelam and Niederkorn, 2017; Juárez et al., 2018). *Acanthamoeba* sp. can also gain access to the bloodstream through the respiratory tract or skin lesions and disseminate to the central nervous system (CNS) causing an often fatal *Acanthamoeba* granulomatous encephalitis (AGE) (Siddiqui and Khan, 2012b; Das et al., 2020; Kalra et al., 2020; Lau et al., 2021). Currently, the development of precise forms of diagnosis and effective therapeutic strategies for reducing the high mortality rates are the main challenges in the control of these amoeba infections (Siddiqui et al., 2016; Anwar et al., 2020; Papa et al., 2020).

Due to their predatory behavior and active phagocytic capacity, FLAs feed on microorganisms to supply their nutritional needs. Thus, by the recently described “feast-fit-fist-feat” theory, *A. castellanii* and other FLAs might have imposed selective pressures on microorganisms. Those that adapted and resisted to the ameboid intracellular environment (Greub and Raoult, 2004; Siddiqui and Khan, 2012b; Goñi et al., 2020) displayed an enhanced expression of virulence factors as well as an increased ability to survive and replicate. These attributes have been associated with their capacity to infect and disseminate into evolutionarily complex hosts and later escalating up to mammals (Casadevall, 2012; Casadevall et al., 2019b; da Silva Ferreira et al., 2021). In this context, *Acanthamoeba* sp. has also functioned as natural reservoirs for epidemiologically important microorganisms, acting as “Trojan horses” for viruses, bacteria, and fungi (Guimaraes et al., 2016).

The association of *A. castellanii* with several fungal pathogens has been documented, amongst which we can highlight: the encapsulated *Cryptococcus neoformans* (*Cn*), the causative agent of the fatal meningoencephalitis in immunosuppressed individuals (Casadevall et al., 2019a; Maliehe et al., 2020); the thermally dimorphic *Histoplasma capsulatum* (*Hc*), extremely resistant to the host’s defense (Guimarães et al., 2011a; Garfoot and Rappleye, 2016), and the commensal *Candida albicans* (*Ca*), often associated with candidemia and invasive candidiasis in immunosuppressed individuals (Boral et al., 2018; Garcia-Rubio et al., 2019).

Several pathogens are able to survive within *A. castellanii* by using similar strategies in the encounter with macrophages (Steenbergen et al., 2001; Van Waeyenberghe et al., 2013; Boral et al., 2018; Albuquerque et al., 2019; Gonçalves et al., 2019; Maliehe et al., 2020); therefore, the main hypothesis points to *Acanthamoeba* sp. and macrophages being evolutionarily related (Siddiqui and Khan, 2012a; Derengowski et al., 2013). *Acanthamoeba* and macrophages share many properties such as cell structure, molecular motility, phagocytosis into vacuoles and production of digestive lysosomal enzymes, physiology, oxidative metabolism, and secretion of extracellular vesicles playing roles on the interaction processes with microorganisms.

Besides these features, the molecular mechanisms involved in the fungal association to both cells seem to be analogous (Garcia-Rubio et al., 2019; Gonçalves et al., 2019; Alcazar-Fuoli et al., 2020). Macrophages recognize fungal cell wall polysaccharides through several pattern recognition receptors (PRRs) inducing phagocytosis, oxidative stress, and pro-inflammatory immune responses (Brown et al., 2018; Kirkland and Fierer, 2020). Among these, mannose receptor (MR) and mannose-binding lectin (MBL) play a key role in fungal recognition by the immune system (Brummer and Stevens, 2010; Goyal et al., 2018; Nikolakopoulou et al., 2020).

Our group pioneered the characterization of two lectin-type receptors, Mannose-Binding Protein and Mannose-Binding Protein-1 (MBP and MPB-1, respectively) on the surface of *A. castellanii*, with the ability to recognize distinct fungal species and thereby mediate a “universal” mechanism of fungus-amoeba interaction (Gonçalves et al., 2019). Both lectins share a domain matching the secondary structure of ConA-like lectins/glucanases superfamily and treatment with soluble mannose specifically inhibited fungal association to trophozoites (Gonçalves et al., 2019). Besides the structural similarity to ConA, no significant similarity to other MBPs were retrieved upon BLAST search.

In this work, we sought to evaluate the importance of MBLs from *A. castellanii* and macrophages by assessing their relative impact on the interactions with fungi and fungal survival. As part of our objective, we performed a targeted search of surface proteins present in *A. castellanii* and murine macrophages to identify possible high affinity ligands and receptors to mannoproteins and fungal cell wall mannans. Therefore, we were able to establish similar relationships between mannose affinity pathways in both phagocytes, which could also evidence the possibility of convergent evolution regarding their interactions with fungal pathogens and fungal adaptation in both models.

## Methods

### Organisms and Growth Conditions

*A. castellanii* ATCC 30234 (American Type Culture Collection, Manassas, VA) was cultured in Peptone- Yeast Extract- Glucose (PYG) () medium (pH 6.5) containing 20 g/L of peptone, 1 g/L yeast extract and 100 mM glucose, supplemented with 0.4 mM CaCl_2_, 0.4 mM MgSO_4_, 2.5 mM Na_2_HPO_4_, 2.5 mM KH_2_PO_4_, 1 g/L sodium citrate, 0.05 mM Fe(NH4)_2_(SO4)_2_) and 1% penicillin/streptomycin (ThermoFisher Scientific, MA, USA) at 28°C (Gonçalves et al., 2019). RAW 264.7 murine macrophages were cultured in Dulbecco Modified Eagle’s Medium (DMEM, pH 7.0) supplemented with 10% heat-inactivated fetal bovine serum, 10% NCTC-109 medium (Sigma-Aldrich, MO, USA), 1% non-essential amino acids (Sigma-Aldrich) and 1% penicillin/streptomycin (ThermoFisher) at 37°C in a 5% CO_2_ incubator. Both cells were cultured until the formation of monolayers for about 48 h and subsequently used in all experiments (Taciak et al., 2018).

Fungi were grown according to standard protocols: *Ca* SC5314 (ATCC MYA 2876) was cultured in Sabouraud medium (pH 5.6) containing 10 g/L of peptone and 40 g/L dextrose (Gácser et al., 2007), *Cn* H99 serotype A (ATCC 208821) in a minimum medium (pH 5.5) composed of glucose (15 mM), MgSO_4_ (10 mM), KH_2_PO_4_ (29.4 mM), glycine (13 mM), and thiamine-HCl (3 μM) (Guimarães et al., 2010) and *Hc* G217B (ATCC 26032), in Ham’s F-12 medium (pH 7.2) supplemented with glucose (18.2 g/L), glutamic acid (1 g/L), HEPES (6 g/L) and cysteine (8.4 mg/L) (Guimarães et al., 2019). All fungi were cultured for 48 h at 37°C with 150 rpm shaking.

### Biotinylation of Phagocytes’ Surface Proteins

*A. castellanii* trophozoites and RAW 264.7 macrophages were grown for 48 h and the percentage of viability was determined by 0.4*%* Trypan Blue dye exclusion, as the number of viable (white) × 100, over total cells (white + blue), with values ~100% for both cells (Strober, 2001). The phagocytes were detached from the culture flasks and washed three times with PBS (Phosphate Buffered Saline pH 8.0;137 mM NaCl, 2.7 mM KCl, 10 mM Na_2_HPO_4_ and 1.8 mM KH_2_PO_4_) at 4°C, with centrifugation at 250*xg* for 10 min for amoebas and 400*xg* for 5 min for macrophages. The cell concentration was adjusted to 25x10^6^cells/mL and surfaces biotinylated by incubating cells with 2 mM Sulfo-NHS-LC-Biotin (ThermoFisher) at 4°C for 30 min (Azim-Zadeh et al., 2007; Gonçalves et al., 2019). The excess of biotin was inactivated by four washes with 100 mM glycine in cold PBS (pH 8.0) and 10 min centrifugation.

### Production of Phagocytes’ Surface Protein Extracts

The cell pellets were resuspended in a cold extraction buffer (0.5% CHAPS; 2 mM β-mercaptoethanol; 25 mM Tris-HCl; 100 mM NaCl; 20 mM CaCl_2_; 1 mM phenylmethylsulfonyl fluoride), with addition of protease inhibitors (Complete Mini Tablets, Roche), and disrupted with a dounce homogenizer and zirconium beads, for 20 cycles in an ice bath, to obtain the total cell extract. The preparations were centrifuged at 18,000*xg* for 30 min at 4°C to obtain the clarified total extracts. The protein concentrations in both extracts were determined by the Pierce™ Bicinchonic Acid (BCA) Protein Assay Kit, ThermoFisher, following manufacturer’s instruction. To analyze the protein pattern of the extracts, 10 μg of proteins were resolved by 10% SDS-PAGE, as described elsewhere (Gonçalves et al., 2019). The efficiency of surface protein biotinylation was analyzed by Western blot using an alkaline phosphatase-streptavidin conjugate (SouthernBiotech, AL, USA) and developed with an NBT/BCIP substrate (ThermoFisher) as described (Gonçalves et al., 2019).

### Affinity of Mannose-Binding Lectins in Phagocyte Surface Protein Extracts

Clarified surface protein extracts of the phagocytes were purified by affinity chromatography assay, using a mannose column, according to manufacturer’s instruction (D-Mannose Agarose, Sigma-Aldrich) (Garate et al., 2004). After washing to remove the preservation solution, the resin was equilibrated with Dulbecco’s Phosphate Buffered Saline (DPBS) with MgCl_2_ and CaCl_2_ (pH 7.4, 0.49 mM MgCl_2_ and 0.90 mM CaCl_2_), with washes at 640*xg* for 5 min. Then, clarified extracts were incubated with the resins, in a proportion of 2.5 mL resin for every 5 mL of clarified extract (2 mg/mL), for 1 h at room temperature. Subsequently, resins were packed, and fractions of unbound proteins (F1) were collected. Then, the columns were washed with 10 bed volume of DPBS, at a 1 mL/min flow to remove non-specific binding proteins (F2). Thereafter, column-bound proteins were eluted with PBS/Ca^+2^/Mg^+2^, plus 2 mM EDTA and 150 mM soluble mannose, and the eluent (F3) was collected in 0.5 mL fractions. The collected fractions F1, F2, and F3 had absorbances monitored at 280 nm, along with quantification by the BCA method, and were evaluated by SDS PAGE and Western blot as described.

### Production of Mannose-Purified Surface Protein Extracts

The mannose-purification of clarified surface protein extracts of *Ac* and RAW 264.7 was scaled up to 96-well microplates previously sensitized with 50 µL of mannose solution (10 µg/mL) for 1 h at 37°C, and subsequently overnight at 4°C. After, the plates were blocked with blocking buffer (SuperBlock™ (PBS) Blocking Buffer, ThermoFisher), for 1 h at 37°C. Thereafter, plates were incubated with 200 µg/mL (50 µL/well) of phagocytes’ clarified extracts in DPBS, for 1 h at 37°C. Posteriorly, plates were washed three times with PBS to remove mannose unbound proteins. Then, mannose-attached proteins were recovered with stripping buffer (50 mM Tris-HCl, 50 mM DTT, and 2% SDS; pH 7.0) for 1 h at 70°C. Next, the content of each well was collected and dialyzed in dialysis cassettes (Slide-A-Lyzer™ 10K MWCO, ThermoFisher) against PBS/Ca^+2^/Mg^+2^ at 4°C, under 200 rpm shaking for three days, with daily buffer exchanges. Then, the mannose-purified surface proteins (MPPs) were collected, identified, and analyzed by Western blot. The protein banding pattern of the MPPs were compared between the distinct phagocytes.

### Identification and Comparison of the Protein Profile of Mannose-Binding Lectins Between Phagocytes

*Ac* and RAW MPPs were processed for analysis by mass spectrometry. We sought to identify and compare the sequences of mannose-binding proteins from both phagocytes to establish any possible degrees of similarity regarding mannose affinity. For isolation of biotinylated surface proteins, the MPPs were purified using the Dynabeads^®^ M-280 Streptavidin Kit with 100 μg of magnetic beads. The beads were washed with PBS + Tween 20 (0.01%) at pH 7.4, separated using a magnet for 5 min and incubated with biotinylated proteins for 30 min at room temperature under orbital shaking. Beads were washed five times with PBS and dehydrated in a speed vac. Beads were resuspended in Urea (7 M) and Thiourea (2 M), added of 1 M HEPES (to a final concentration of 100 mM), followed by 100 mM dithiothreitol (to 10 mM), and incubated for 1 h at 30°C. Iodoacetamide (400 mM) was added to a final concentration 40 mM, with subsequent incubation for 30 min in the dark. Then, 175 μL of ultrapure H_2_O TEDIA and trypsin solution in 0.1 M acetic acid (0.1 μg/μL were added to a final concentration of 16 ng/μL and incubated overnight at 37°C. After this incubation, 2 μL of 10% TFA trifluoroacetic acid (TFA) (0.1% final concentration) were added. Then, peptides were purified in an UltraMicroSpin™columns (Havard Apparatus) and dried in a speed vac. At the end of the process, tryptic peptides were solubilized in 10 μL of 0.1% TFA and about 4 μL of the mixture wwere loaded onto an in‐house packed column (15 cm × 75 μm) filled with 3 μm ReproSil C_18_ resin (Dr. Maisch GmbH) using the Nano LC-Ultra nano liquid chromatography system (Eksigent Technologies) (Gonçalves et al., 2018).

### Mass Spectrometry Analysis

An amount of 0.8 μg of peptides was injected in a nano-LC–MS/MS system consisting of an EASY II-nano LC system (Proxeon Biosystem, Denmark), for LC-based separation, coupled to a nano-ESI LTQ-Orbitrap Velos mass spectrometer (Thermo Fisher). Peptide separation was performed in NanoAccquity system equipped with C-18 ReproSil 5 μm resin (Dr. Maisch) and a New Objective PicoFrit^®^ analytical column (75 μm × 20 cm) packed with Reprosil-pur C18-AQ 3 μm resin (Dr. Maisch). For each sample, peptides elution used a linear gradient of solvent solution ACN 95%, 0.1% TFA from 5% to 20% for 85 min, 20%-40% for 22 min, 40%-95% for 5 min and 95% for 8 min, using a flow rate of 250 nL/min. Mass spectra were acquired in a positive mode top 10 DDA (Data-Dependent Acquisition) method. MS1 scan was acquired in an Orbitrap analyzer set for a 350-1800 m/z range, 60000 resolution (at m/z 400) with a minimal signal required of 10000, isolation width of 2.0. The 10 most intense ions were fragmented by collision-induced dissociation (CID) at 30 NCE (Normalized Collision Energy) with a dynamic exclusion of 30s. The acquired spectra were processed using the peptides search engine BSI PEAKS^®^ X+ 10.5 (Bioinformatics Solution Inc.) for peptide identification. The considered parent mass error tolerance and fragment mass error tolerance were both set to 0.6 Da and 0.5 Da, with precursor mass search type monoisotopic. The maximum missed tryptic cleavages was set to two, and one non‐specific cleavage was allowed. Carbamidomethylating was set as fixed modification (MW = 57.02 Da) and as variable modifications, methionine oxidation (MW = 15.99 Da) and lysine Sulfo-NHS-LC-Biotin conjugation (MW = 339.16 Da) were allowed. The *A. castellanii* trophozoites (14,944 proteins) and *Mus musculus* (86,586 proteins) databases were downloaded at www.uniprot.org as for April 2021. The considered false discovery rate was set at 1%. The mass spectrometry proteomics data have been deposited to the ProteomeXchange Consortium *via* the PRIDE (Perez-Riverol et al., 2019) partner repository with the dataset identifier PXD031121.

### *De Novo* Sequencing Analysis

An output of *de novo* peptides was obtained from the Peaks^®^X+ software and analyzed in the DeNovo Sequencing (PepExplorer) of the PatternLab for Proteomics (http://www.patternlabforproteomics.org). The PepExplorer was set up against the same target-decoy sequence database of *Ac* and RAW as described above, according to instructions informed elsewhere (da Veiga Leprevost et al., 2015), with a set of parameters that were adjusted to optimize the analysis process and most stringent comparison parameters, including the ReverseDecoy Label insertion, minimal identity of 0.7, a minimum peptide size of 5 amino acids (for *Ac*), minimal identity of 0.8, a minimum peptide size of 5 amino acids (for *M. musculus*), and a denovo cut off score of 90, for both. After analysis, the dynamic report showed a list of identified proteins and the corresponding peptides based on sequence alignments that were compared between samples for the determination of proteins with affinity to mannosylated residues. Proteins abundances were calculated according to the number of spectra of all peptides identified and validatedby *de novo* sequencing a specific protein divided by the sum of all counted spectra for each of the candidate proteins (Ishihama et al., 2005; Guimarães et al., 2011b). Further proteins annotation and gene ontology molecular function determination were performed using the online DAVID database for annotation (https://david.ncifcrf.gov). To detect similarities between *A. castellanii* and *M. musculus* MPPs sequences identified, we performed a BLASTp search (Johnson et al., 2008) with the proteins identified in **Supplementary Table 1** as queries. The results were filtered by selecting the macrophage identified MPPs (**Supplementary Table 2**) with a percentage of identity greater than 30% and suitable maximum score (>30).

### Binding of Phagocytes Surfaces Protein Extracts to Mannose and Mannan

An indirect ELISA was used to evaluate the binding of clarified surface protein extracts of phagocytic cells to mannose and mannan. Initially, 96-well plates were coated with mannose or mannan as described and blocked with blocking solution (1% BSA in TBS-T) for 1 h at 37°C. After three washes, the biotinylated clarified extracts were serially diluted (200 to 0.09 µg/mL) in blocking solution and added to wells for 1 h at 37°C. Non-biotinylated clarified extracts of both phagocytes were used as negative controls. Thereafter, the plates were washed three times and incubated with an alkaline phosphatase-streptavidin conjugate (SouthernBiotech) diluted at 0.5 µg/mL in blocking solution, for 1 h at 37°C. The wells were washed three times with TBS-T and reactions developed with p-nitrophenyl phosphate (pNPP tablets, Sigma-Aldrich), at 37°C for 15 to 30 min. The absorbances were read at 405 nm on a microplate reader (Molecular Devices, CA, USA) (Guimarães et al., 2009).

Likewise, MPPs binding performances were evaluated under similar indirect ELISA, as previously described, and reactions compared between both phagocytes and to clarified total extracts.

### Binding of Mannose-Purified Proteins to Fungi

Several techniques were used to characterize the binding of the MPPs from both phagocytes to fungi, including indirect ELISA, flow cytometry, and fluorescence microscopy Guimarães et al., 2009; (Guimarães et al., 2010; Cordero et al., 2016; Gonçalves et al., 2019). For indirect ELISAs, yeasts were washed three times with PBS at 1,100 x g for 5 min, fixed in 4% paraformaldehyde for 30 min at room temperature, resuspended in PBS and enumerated. After, plates were coated (10^6^ yeasts/well) and blocked with blocking solution for 1 h at 37°C. Subsequently, plates were incubated with the serially diluted MPPs of both phagocytes, alkaline phosphatase-streptavidin conjugate, and upon incubation with substrate, absorbances were measured as previously described. Non-biotinylated clarified extracts were used as negative controls. For the flow cytometry and fluorescence microscopy assays, yeasts were washed three times and blocked with 2% BSA in PBS for 2 h at room temperature with shaking. Upon washes, 10^7^ yeasts were incubated with clarified or MPPs (100 µg) diluted in 1 mL PBS/Ca^+2^/Mg^+2^ plus 2% BSA and incubated for 2 h at room temperature. After three washes with PBS/Ca^+2^/Mg^+2^, yeasts were incubated with the streptavidin-Alexa 488 conjugate (ThermoFisher) at 5 μg/mL blocking solution for 1 h at room temperature, in the dark, and subsequently washed three times. As a positive control for the binding to mannosylated components on the fungal cell wall, yeasts were incubated with ConA-TRITC (at 5 μg/mL; Thermo Fisher) for 1 h at room temperature, in the dark. After washing, yeasts were incubated with 0.5 µg/mL of Uvitex 2B (Polyscience, USA) for 30 min at room temperature in the dark, with three further washes with PBS/Ca^+2^/Mg^+2^. The yeasts were examined using an Axio Imager microscope (Carl Zeiss MicroImaging Inc., USA), using a 495 nm filter, appropriate for the fluorochrome used, at 100x. In addition, the preparations were analyzed in a FACSCalibur (BD Biosciences, USA) and the intensity of FL1^+^ cells (for binding to streptavidin-Alexa 488) and FL2^+^ cells (for binding to ConA-TRITC) were recorded, Yeasts were also incubated with either only non-biotinylated clarified extracts or fluorophore conjugates, as negative controls (Gonçalves et al., 2019) and the average fluorescence intensity compared among groups.

### Cross-Inhibition of the Interaction Between Phagocytes and Fungi

To confirm the similarity of recognition to fungal mannosylated residues by phagocytic cells, their crossed-inhibiting effect on fungal interaction was evaluated. *Ac* and RAW were plated at a concentration of 4x10^5^ cells/well in 24-well plates and incubated overnight under specific conditions. Yeasts were washed with PBS and labeled with 0.04 mg/ml NHS-Rhodamine (Thermo Fisher) diluted in PBS, for 1 h at room temperature. After five washes, the yeasts were pre-incubated with 50 µg/mL of MPPs from either phagocyte for 1 h at room temperature. Following three washes with PBS, yeast concentrations were adjusted and added to phagocytes at a multiplicity of infection (MOI) of 5 fungi: 1 phagocytic cell in a crossed-way: (i) yeasts pre-incubated with MPPs of RAW 264.7 interacted with amoeba and (ii) yeasts pre-incubated with MPPs of *Ac* interacted with macrophages, for 30 min and 2 h, under appropriate conditions for each phagocytic cell. The stipulated interaction times were based on information from live-microscopy experiments, for early adhesion and late interaction (Gonçalves et al., 2019). After, phagocytes were washed three times, detached from the plate by pipetting up and down and analyzed in a FACSCalibur (BD Biosciences). The interaction ratio (i.e., FL2^+^ phagocytes interacting with fungi, over the total number of cells analyzed) was calculated:


$$FACS\;Interaction\;ratio\left(\phi h\right):\frac{\left(number\;of\;interacting\;phagocytes\right)*100}{total\;number\;of\;phagocytes}$$

### Evaluating the Importance of the Mannose Receptor on the Phagocyte–Fungi Interaction

Inhibition experiments were performed to confirm the importance of MBPs from each specific phagocyte model during phagocytosis. Phagocytes were plated (4x10^5^ cells/well) in 24-well plates under the appropriate growth conditions. Yeasts were washed, NHS-Rhodamine stained as described, and diluted in the required media for each phagocyte (PYG or DMEM) with or without 100 mM soluble mannose, at a MOI of 5 fungi: 1 phagocytic cell. Plates were incubated for 30 min and 2 h (Gonçalves et al., 2019) under best condition for each phagocyte and washed three times. The intracellular yeasts that remained adhered to the phagocytes were stained with 0.5 µg/ml of Uvitex 2B. Therefore, it was possible to distinguish the internalized yeasts, displaying a red fluorescence, whereas yeasts adhered to the phagocyte surface, displayed simultaneously red and blue fluorescence. Phagocytes were detached from the plates, fixed, and analyzed by flow cytometry. Additionally, the cells were examined under a fluorescence microscope at 63x objective magnification. Through the records and cell counts of each experimental group, several parameters were calculated (Gácser et al., 2007; Guimarães et al., 2019; Cseresnyes et al., 2020), including:


$$\text{Interaction}\;\text{ratio}\;(\phi h):\frac{\left(\text{number}\text{of}\;\text{interacting}\;\text{phagocytes}\right)}{\left(\text{total}\;\text{number}\;\text{of}\;\text{phagocytes}\right)}$$

$$Adhesion\;ratio\;\left(\phi adh_{ratio}\right):\frac{\left(number\;of\;adhered\;yeasts\right)}{\left(number\;of\;adhered\;yeasts+number\;of\;internalized\right)}$$

$$Internalization\;ratio\;\left(\phi int_{ratio}\right):\frac{\left(number\;of\;internalized\;yeasts\right)}{(number\;of\;adhered\;yeasts+number\;of\;internalized})$$

$$Symmetrized\;adhesion\;index\;\left(\phi adh_{sym}\right):\phi adh_{ratio}\mathrm{X\phi}h$$

$$Symmetrized\;internaliztion\;index\;\left(\phi int_{sym}\right):\phi int_{ratio}\mathrm{X\phi}h$$

The Interaction ratio (Φh) describes the ratio of host cells that has at least one interacting pathogen. The adhesion (Φadh_ratio_) and internalization (Φint_ratio_) ratios describe respectively the proportion of adhered and internalized fungi relative to the total number of interacting fungi. Symmetrized adhesion (Φadh_sym_) and internalization (Φint_sym_) indices reveal the overall probability of a fungus to be respectively adhered or internalized by a host cell. All parameters were compared between the mannose treated versus untreated and between both phagocytes for early adhesion and late interaction events.

### Assessing the Importance of the Mannose Receptor for Fungal Viability

Fungal viability was assessed by determining colony forming units (CFUs) upon interaction inhibition with mannose. Phagocytic cells were plated (4x10^5^ cells/well), incubated overnight, and yeast added at a MOI of five fungi: one phagocyte in the presence or absence of 100 mM soluble mannose. Initially, interactions occurred for 2 h to allow phagocytosis of the majority of yeasts, as described in previous procedures (Gonçalves et al., 2019). Upon incubations, systems were washed three times with PBS, the appropriate culture media for each phagocyte was added and the plates returned to the appropriate conditions of either phagocytic cells, remaining for additional 24 h. Subsequently, the phagocytes were lysed with cold sterile H_2_O for 1 h, followed by 15 to 20 passages through a 26 G needle and recovered yeasts plated on specific media and kept at the appropriate temperature conditions for either yeasts (Cordero et al., 2016; Gonçalves et al., 2019; Guimarães et al., 2019).

In addition, fungal viability upon interactions was also assessed by fluorescence microscopy. Yeasts were incubated with two-color fluorescent viability probe FUN™ 1 (10 mM, ThermoFisher), diluted in 1 mL of glucose buffer (2% glucose, 10 mM HEPES; pH 7.2), for 30 min at 37°C, in the dark (Gantner et al., 2005; Gácser et al., 2007; Slesiona et al., 2012). After washes with glucose buffer, yeasts were incubated with phagocytes, in the absence and presence of 100 mM mannose, as previously described. Yeasts that remained adhered to the phagocyte surface were labeled with Uvitex 2B. Therefore, internalized yeasts displayed a red (viable) or yellow-green (non-viable) fluorescence, whereas yeasts adhered to the phagocyte surface displayed a red (viable) or yellow-green (non-viable) and a blue fluorescence. Analyses were carried out by examining the preparations in an Axio Imager microscope at 63x magnification. Cell counts of each experimental group, in different fields, were performed and the following parameters were determined (Cseresnyes et al., 2020).


$$Viabilityratio\left(\phi viable_{ratio}\right):\frac{\left(numberofviableyeasts\right)}{\left(numberofviableyeasts+numberofdeadyeasts\right)}$$

$$Mortalityratio\left(\phi dead_{ratio}\right):\frac{\left(numberofdeadyeasts\right)}{\left(numberofviableyeasts+numberofdeadyeasts\right)}$$

### Statistical Analysis

Statistical analyses were performed using GraphPad Prism version 8.00 for Windows (GraphPad Software, La Jolla California USA, www.graphpad.com). Comparisons between three or more groups were made by two-way ANOVA, with subsequent Tukey’s or Dunnett’s multiple comparisons test. Comparisons between two groups were made using the Student *t-*test. Values of p ≤ 0.05 were considered statistically significant. Each experiment was repeated at least three times.

## Results

### Extracts Purified With Mannose Display Distinct Protein Complexity Between Phagocytes

**Figure 1:**
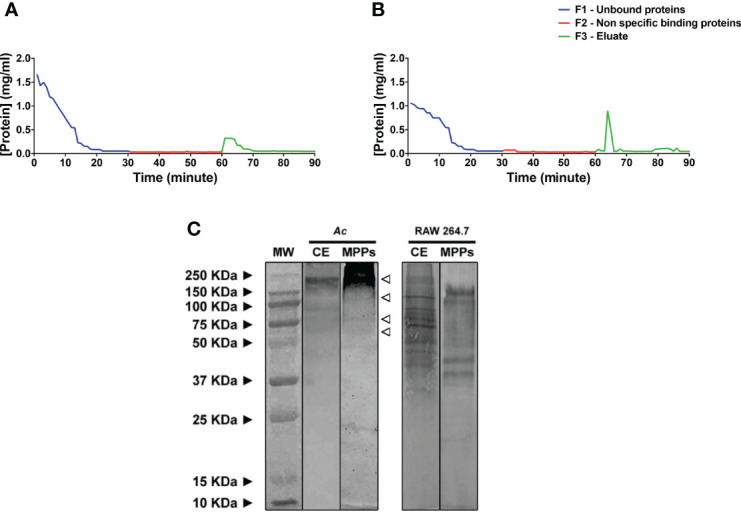
Differences between the profile composition of mannose-binding lectins on the surface of *Acanthamoeba castellanii* and macrophages. **(A, B)** Affinity chromatography with D-mannose agarose columns displaying the chromatogram results upon purification of surface extracts of **(A)** *A. castellanii (Ac)* and **(B)** RAW 264.7 macrophages. **(C)** Western blot, corresponding to clarified and (MPPs, of *Ac* and macrophages. The molecular weight standard was used in the analysis. Lane MW: Molecular Weight Standard (Prestained™ Standards, Kleidoscope™); Lane *Ac* CE: Clarified extract of *Ac*; Line *Ac* MPPs: MPPs of *Ac*, with open arrows indicating the molecular weight of approximately 55 KDa, 75 KDa, 110 KDa and above 150 KDa; Lane RAW 246.7 CE: Clarified extract of RAW 246.7 macrophages; Lane RAW 246.7 MPPs: MPPs of RAW 246.7 macrophages where nine bands were identified, ranging from 37 KDa to 150 KDa.

The clarified extracts of both phagocytes were purified using a mannose-coupled resin, as described elsewhere (Garate et al., 2004), and both chromatograms display the purification efficiency and isolation of proteins with affinity to mannose (**Figures 1A, B**). For the purification of amoeboid extracts, the eluate (F3 fraction) comprised the fractions 2-10 and displayed concentrations ranging from 0.32 to 0.16 mg/mL, with the amount of total proteins that bound to mannose consisting of 12.9% of the total extract (**Figure 1A**). Eluted fractions (F3) from the purification of macrophage extracts localized at the fractions 4 and 5, with concentrations of 0.9 and 0.5 mg/ml respectively, with higher values when compared to ameboid extracts, and recovered mannose-binding proteins totalized 17.3% of the total protein mass (**Figure 1B**). Altogether, differences in protein concentration and elution time of MPPs between the phagocytes suggest higher relative content or greater affinity of the mannose-binding lectins in macrophage extracts relative to those in the ameboid extracts.

Upon a large-scale purification/enrichment of MPPs, Western blot of purified extracts of *Ac* revealed four mannose-affinity proteins of different molecular weights - approximately 55 KDa; 75 KDa; 110 KDa and above 150 KDa, whereas in macrophages, nine bands were identified, ranging from 37 KDa to 150 KDa (**Figure 1C**). Differences observed in the complexity of protein patterns between phagocytes point to greater diversity and variability of mannose-binding lectins on the surface of macrophages. However, upon purification, the presence of bands around 55 KDa, 75 KDa, and 100 KDa in similar ways was also evidenced in the two phagocytes.

### Phagocytes’ Biotinylated Extracts Showed a Dose-Dependent Binding to Mannose and Mannan

**Figure 2:**
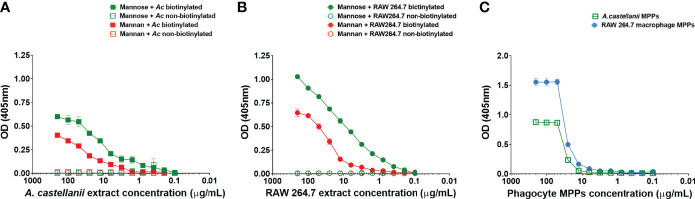
*Acanthamoeba castellanii* and macrophage extracts showed a dose-dependent binding to mannose and mannan. Indirect ELISA demonstrated dose dependent binding of clarified extracts of **(A)** *A. castellanii (Ac)* and **(B)** RAW 264.7 macrophages to mannan and mannose. **(C)** MPPs of *Ac* and macrophages displayed a more intense binding to mannose than the phagocyte’s biotinylated surface extracts. In addition, MPPs of macrophages seem to bind more effectively to mannose in comparison to *Ac* MPPs. The streptavidin-alkaline phosphatase conjugate was used to detect biotinylated proteins. As a negative control, clarified non-biotinylated extracts were used in the process and displayed no reactivity.

The indirect ELISA assays revealed a dose-dependent binding to mannose with concentrations above 0.78 μg/mL for clarified biotinylated surface extracts of amoeba, in contrast to concentrations above 0.20 μg/mL for macrophages (**Figures 2A, B**). Nevertheless, the clarified RAW macrophages’ extracts (**Figure 2B**) overall displayed higher absorbances than *Ac* extracts (**Figure 2A**), configuring higher affinity.

Upon purification, the evaluation of MPPs’ affinity to mannose (**Figure 2C**) displayed detectable binding above 12.5 μg/mL for proteins of amoeba origin, in comparison to 3.1 μg/mL for macrophages’ proteins; however, MPPs preparations of both phagocytes displayed saturation at 50 μg/mL, despite the similar K_d_ values for both extracts (K_damoeba_= 59.15 and K_dmacrophage_= 51.44). The maximum binding capacity was higher for macrophages (Bmax_macrophage_= 1.55) versus *Ac* MPPs (Bmax_amoeba_= 0.88), confirming the higher affinity.

### Phagocytes Extracts Binding to Fungal Cell Wall Components

**Figure 3:**
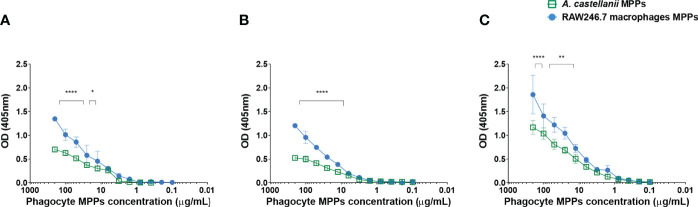
*Acanthamoeba castellanii* and macrophages mannose-purified proteins (MPPs) showed a dose-dependent binding to fungi. Indirect ELISA to analyze the binding of MPPs of *A. castellanii* and RAW 264.7 macrophages to **(A)** *Candida albicans*, **(B)** *Cryptococcus neoformans* and **(C)** *Histoplasma capsulatum*. (*p < 0.05; **p < 0.001 and ****p < 0.0001).

Amoeba and macrophage MPPs displayed a dose-dependent binding to all three fungi of distinct cell wall structure tested (**Figure 3**, p<0.05). Corroborating with the aforementioned results, macrophage MPPs exhibited greater binding intensity when compared to the ameboid MPPs to *Ca* (**Figure 3A**), *Cn* (**Figure 3B**) and *Hc* (**Figure 3C**), pointing to greater affinity of MPPs present on the macrophage surface.

### Binding Pattern of Phagocytes’ MPPs to Fungal Cell Surface

**Figure 4:**
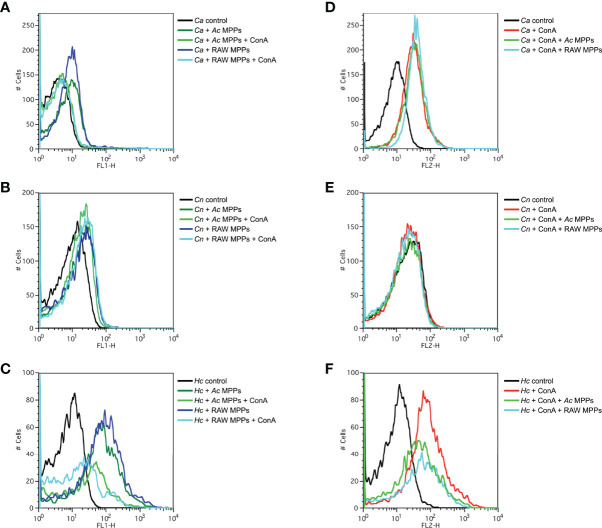
Mannose-purified proteins (MPPs) of *Acanthamoeba castellanii* and macrophages recognized targets on the fungal cell wall. **(A–C)** Histograms representing the FL1-H fluorescence and binding intensity of phagocytes MPPs to fungi **(A)** *Candida albicans* (*Ca*), **(B)** *Cryptococcus neoformans* (*Cn*) and **(C)** *Histoplasma capsulatum* (*Hc*), with the respective co-incubation with Concanavalin A (ConA) for inhibition evaluations. **(D–F)** Besides, yeast labeling with the positive control ConA data on **(D)** *Ca*, **(E)** *Cn* and **(F)** *Hc*, which were analyzed by FL2-H intensity, are demonstrated, along with respective incubations with the MPPs of each phagocyte for inhibition evaluation.

The binding intensity and qualitative pattern of the MPPs of *Ac* and RAW macrophages to fungi were analyzed by flow cytometry and fluorescence microscopy and compared with ConA-TRITC mannose-binding lectin (**Figure 4** and **Supplementary Figure S1**). The means of fluorescence intensity of yeasts incubated with MPPs of either origin differed significantly from their respective negative controls (**Figure 4A**, p<0.0001; **Figure 4B**, p<0.01; **Figure 4C**, p<0.0001), and demonstrated a similar profile to the positive ConA control, which bound significantly more to the surface of *Ca* and *Hc* (**Figure 4D**, p<0.0001; **Figure 4F**, p<0.0001), and weakly to *Cn* (**Figure 4E**). Phagocytes’ comparison, therefore, demonstrated that macrophage MPPs bound more efficiently than amoeba MPPs (**Figures 4A–C**). Confirming the binding to the same target, addition of ConA inhibited the binding of both MPPs in all instances (light curves, **Figures 4A–C**); however, addition of either MPPs had a statistically significant impact on ConA binding only in the case of *Hc* (**Figure 4F**).

**Figure 5:**
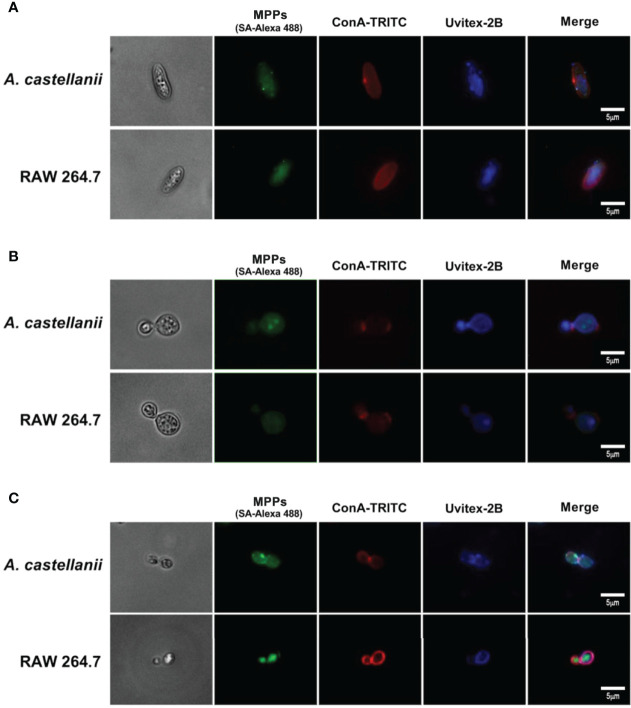
Mannose purified proteins (MPPs) of *Acanthamoeba castellanii* and RAW 246.7 macrophages co-localized with a mannose-specific lectin Concanavalin A. Fluorescence microscopy images demonstrate the cell wall of the fungi **(A)** *Candida albicans*; **(B)** *Cryptococcus neoformans* and **(C)** *Histoplasma capsulatum* stained in blue (Uvitex 2B); MPPs of phagocytes stained in green upon incubations with yeasts, followed by the streptavidin-Alexa 488 conjugate; and the mannosylated components of the yeast cell wall stained in red, after incubations with ConA-TRITC. Scale bar= 5μm.

Besides, it is possible to observe similarities in the patterns of binding of MPPs as a punctuated way to along the external layer of the cell walls of *Ca* (**Figure 5A**), *Cn* (**Figure 5B**) and *Hc* (**Figure 5C**), which co-localized, at the cell wall level with mannosylated regions marked by ConA and chitin by Uvitex2B.

### Binding Nature of Phagocytes’ MPPs to Fungi

**Figure 6:**
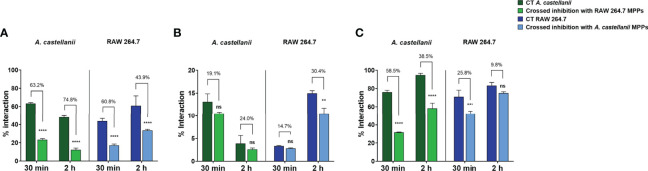
*Acanthamoeba castellanii* and RAW mannose-binding lectins share the same binding targets on the fungal cell wall. **(A)** *Candida albicans*, **(B)** *Cryptococcus neoformans* and **(C)** *Histoplasma capsulatum* yeasts were pre-incubated with 50 µg/mL of either *A. castellanii* or RAW 264.7 MPPs. After this stage, cross-inhibitions were evaluated, where yeasts pre-treated with RAW 264.7 MPPs interacted with *A. castellanii* (light green bar) and compared to controls in the absence of inhibition (dark green bar). Additionally, yeasts pre-treated with *A. castellanii* MPPs interacted with RAW 264.7 macrophages (light blue bar), and compared to controls in the absence of inhibition (dark blue bar). Interactions were performed for 30 min and 2 h, to evaluate the initial fungal adhesion and the late interactions, respectively. Inhibition percentages (numbers above bars) were calculated from the interaction percentages as follows: (Control - Pre-treated group) x 100/Control. The statistical significances comparing inhibition x controls are represented above each group (**p < 0.01; ***p < 0.001; ****p < 0.0001; ns, not significant). The bars represent the average of three independent experiments, performed in triplicates.

Upon the confirmation of binding to mannose/mannan or yeasts´ surface by the proteins present in MPPs of *Ac* and RAW, cross-inhibitions were performed to evaluate whether, in fact, MPPs from either *Ac* and RAW bind to the same target on the fungal cell wall. The pre-incubation of yeasts with MPPs of either phagocyte inhibited the fungus-second phagocytic cell interaction, at early (30 min) and late (2 h) events (**Figure 6**). Pre-incubations with macrophage MPPs inhibited the early association (at 30 min) of *Ac* with *Ca* (63.2% inhibition, p<0.0001; **Figure 6A**) and *Hc* (58.5% inhibition, p<0.0001; **Figure 6C**). Similarly, pre-incubations with *Ac* MPPs were able to impact the association of macrophages with *Ca* (60.8% inhibition; p<0.0001; **Figure 6A**) and *Hc* (25.8% inhibition; p<0.001; **Figure 6C**). For 2 h of interaction, pre-incubations with macrophage MPPs inhibited the *Ac* engulfment of *Ca* (74.8%, p<0.0001; **Figure 6A**) and *Hc* (38.5%, p<0.0001; **Figure 6C**) by amoeba, whereas pre-treatment with *Ac* MPPs inhibited the interaction between macrophages and *Ca* (43.9%, p<0.0001; **Figure 6A**) and *Cn* (30.4%, p<0.01; **Figure 6B**). The occurrence of inhibition in the two phagocytic models suggests binding of MPPs of both phagocytes to the same targets of the fungal cell wall.

### The Recognition of Mannosylated Residues Is Important for the Phagocytes-Fungi Interaction

**Figure 7:**
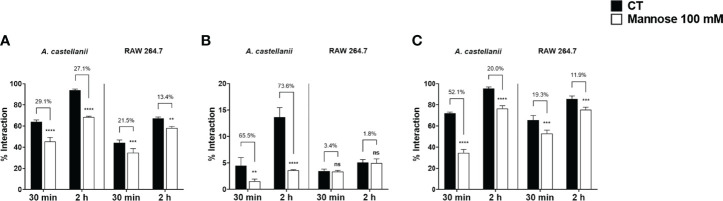
The mannose receptor plays an important role in the events of fungal interaction with *Acanthamoeba castellanii* and macrophages. After NHS-Rhodamine labeling of **(A)** *Candida albicans*, **(B)** *Cryptococcus neoformans* and **(C)** *Histoplasma capsulatum*, interaction assays were performed with both *A. castellanii* and RAW 264.7, with phagocytes model indicated on the graphs, in two conditions: absence (Control group, black bars) and presence of 100 mM soluble mannose (Mannose 100 mM, white bars) and analyzed upon 30 min and 2 h. The interaction ratios were determined in each case, and inhibition percentages (numbers above bars) calculated as followed: (Control - Pre-treated group) x 100/Control. The statistical significances comparing inhibition x controls are displayed above each group (**p < 0.01; ***p < 0.001; ****p < 0.0001; ns, not significant). The bars represent the average of three independent experiments, performed in triplicates.

**Figure 8:**
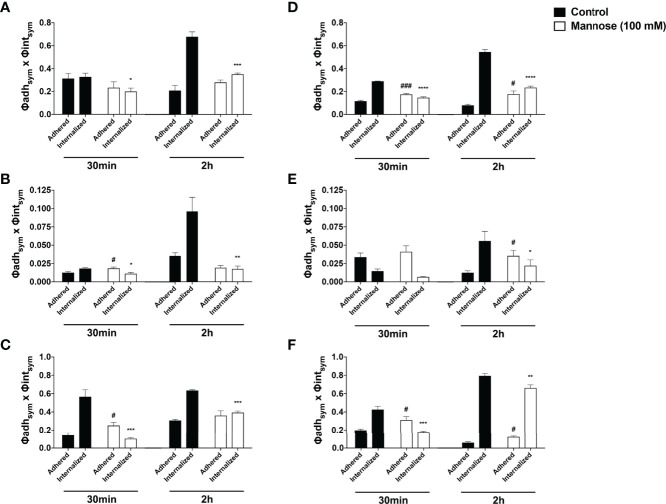
The mannose receptors participates on fungal internalization by *Acanthamoeba castellanii* and macrophages Phagocytosis assays were performed with either **(A–C)** *A. castellanii* or **(D–F)** RAW 264.7 upon addition of NHS-Rhodamine labeled **(A, D)** *Candida albicans*, **(B, E)** *Cryptococcus neoformans* and **(C, F)** *Histoplasma capsulatum*, in the absence (Control group, black bars) and presence of 100 mM soluble mannose (Mannose 100 mM, white bars) and analyzed after 30 min, to evaluate the initial fungal adhesion, and 2h, to evaluate the late interaction. Symmetrized adhesion and phagocytic indices were determined and the mannose inhibition groups were compared to controls. The statistical significances are represented above each group, where *p < 0.05, **p < 0.01, ***p < 0.001 or ****p < 0.0001 indicate statistically significant decrease upon mannose inhibition; ^#^p < 0.05, ^###^p < 0.001 indicate statistically significant increase upon mannose inhibition; ns = not significant). The bars represent the average of three independent experiments, performed in triplicates.

To clearly determine the impact of MBPs on the phagocyte-fungi interaction, we further performed the interaction assays for 30 min and 2 h using 100 mM soluble mannose as an inhibitor. The results were obtained through analysis by flow cytometry and fluorescence microscopy to establish within the interaction the probability of adhesion and internalization events (**Figures 7**, **8**). The presence of mannose inhibited the interaction between phagocytic cells and yeasts, at all times analyzed. At 30 min of interaction, inhibition of association with *Ac* was observed for *Ca* (29.1%; p<0.0001, **Figure 7A**), *Cn* (65.5%; p<0.05, **Figure 7B**) and *Hc* (52.1%; p<0.0001, **Figure 7C**). For late events (2 h), mannose also had an impact on the interaction of *Ac* with *Ca*, *Cn*, and *Hc* (27.1%, 73.6%, and 20.0% inhibition, respectively; p<0.0001). Regarding macrophages, within the first 30 min, soluble mannose affected RAW-fungi association, to *Ca* and *Hc* (21.5% and 19.3% inhibition, respectively; p<0.001). At 2 h, mannose inhibition was less impacting, but it still impaired the phagocytosis of *Ca* and *Hc* (13.4%, **Figure 7A** and 11.9% inhibition, **Figure 7C**, respectively; p<0.01). Mannose was not able to inhibit the interactions of *Cn* and RAW macrophages at the two time-points evaluated (**Figure 7B**). These observations suggest the participation of mannose-binding lectins on the phagocytes-yeast interaction, and blocking these receptors affects both early fungal adhesion and phagocytosis. Overall, we can conjecture a greater inhibitory effect by mannose in the interaction between fungi and *Ac* than macrophage, and possibly, MBPs are essential and exclusive pathways for the interaction of amoeba with pathogens, whereas in the context of interaction with macrophages, mannose receptors have less expressive action or there is a compensation, with the participation of other pathways for fungal recognition.

To dissect the impact of the mannose dependency on the events of fungal adherence and internalization to phagocytes, the samples were examined under a fluorescence microscope and symmetrized indices of adherence and internalization were determined, i.e., the overall probability of any fungi to be adhered or internalized by a phagocytic cell (Gácser et al., 2007; Guimarães et al., 2019) (**Figure 8**). For the *Ac*-fungi interaction upon 30 min, the presence of soluble mannose seemed to directly impair the adhesion and initial internalization of *Ca*; at 2 h, despite similar average of adhered fungi, the mannose greatly inhibited the overall internalization of *Ca* (**Figure 8A**). For *Cn*, a higher number of adhered yeasts along with less internalization were observed under mannose inhibition at 30 min; however, at 2 h, mannose inhibition seems to impair both adherence and internalization process of *Cn* by *Ac* (**Figure 8B**). For *Hc* at 30 min of interaction, soluble mannose impaired initial internalization, resulting in higher number of adhered yeasts; therefore at 2 h, even with similar average number of attached yeasts per *Ac*, the internalization process is greatly impaired (**Figure 8C**). Overall, inhibition with mannose also resulted in a disbalance, with greater adhesion and lower fungal internalization by macrophages at 30 min; within 2 h, mannose inhibition greatly impacted the internalization of *Ca* (**Figure 8D**), *Cn* (**Figure 8E**) and *Hc* (**Figure 8F**) by RAW macrophages, with more adhered yeast as a result. Altogether, these findings demonstrate that mannose blocks the early fungal entry through high-affinity receptors, which constitute faster pathways of interaction and for the initial adhesion/encompassing phase. Furthermore, mannose inhibition continues to impact the late interaction mechanisms and the action of lower affinity receptors in the establishment of posterior connections for the phagocytic process.

### Mannose Receptor Affects Fungal Survival in the Intracellular Environment of Phagocytes

**Figure 9:**
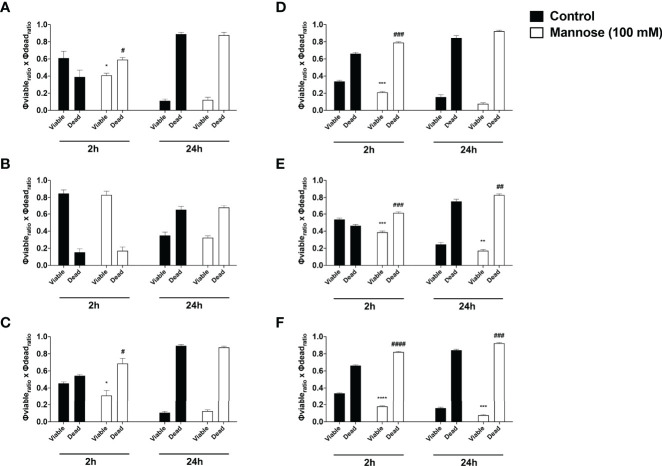
Inhibition of mannose receptor impacts fungal survival inside *Acanthamoeba castellanii* and RAW macrophages. Fungal survival upon interactions with either **(A–C)** *A. castellanii* and **(D–F)** RAW 264.7 macrophages was evaluated for **(A, D)** *Candida albicans*, **(B, E)** *Cryptococcus neoformans* and **(C, F)** *Histoplasma capsulatum* were performed in the absence (Control group, black bars) and presence of 100 mM soluble mannose (Mannose 100 mM, white bars), and viability and mortality ratios were determined. The Mannose groups were compared to controls. Statistical differences are represented above each group (*p < 0.05, **p < 0.01, ***p < 0.001 or ****p < 0.0001 indicate statistically significant decrease upon mannose inhibition; ^#^p < 0.05, ^##^p < 0.01, ^###^p < 0.001 or ^####^p < 0.0001 indicate statistically significant increase upon mannose inhibition; ns, not significant). The bars represent the average of three independent experiments, performed in triplicates.

As demonstrated in competition trials with soluble mannose, the mannose binding proteins play an essential role in the adhesion and internalization of fungi by phagocytes. However, we also assessed the impact of the mannose recognition on the viability of internalized fungi by monitoring the viability and mortality ratios (Gantner et al., 2005; Gácser et al., 2007; Slesiona et al., 2012) (**Figure 9**). Upon 2 h, co-incubations with mannose reduced the viability ratios for *Ca* and *Hc* in comparison to the control groups in the *Ac*-fungi interaction (**Figures 9A, C**, respectively). For these two fungi, similar results upon interactions with RAW macrophages were observed. Instead, for *Cn*, the inhibition of the mannose receptor had no impact on the proportion of alive/dead yeasts in the interactions with *Ac* (**Figure 9B**) in contrast to RAW macrophages, in which blockade of mannose receptors seemed to reduce fungal viability. Altogether, the inhibition assays highlight the role of the mannose receptor on fungal entry and survival.

**Figure 10:**
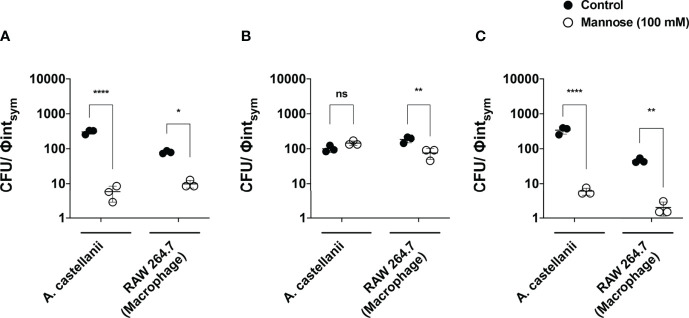
The mannose receptor plays an important role in fungal survival inside *Acanthamoeba castellanii* and RAW macrophages. The phagocyte interaction assays with **(A)** *Candida albicans*, **(B)** *Cryptococcus neoformans* and **(C)** *Histoplasma capsulatum* were performed in the absence (solid circles) and presence of 100mM soluble mannose (empty circles), and fungal viability was assessed by determining the number of Colony Forming Units (CFUs) 24 h upon interaction to assess the fungicidal capacity of phagocytes (*p < 0.05, **p < 0.01 or ****p < 0.0001 indicate statistically significant decrease upon mannose inhibition; ns, not significant). The data represent the average of three independent experiments, performed in triplicates.

Given the results exposed and the likelihood of mannose inhibition to impact on fungal survival in the intracellular environment of phagocytes, we assessed the fungicidal capacity of phagocytes by determining the colony-forming units (CFUs), at 24 h upon internalization. The absolute CFU numbers obtained were normalized by the symmetrized internalization (phagocytic) index, to estimate relatively the number of viable pathogens that were internalized by phagocytes. For *Ca* (**Figure 10A**) and *Hc* (**Figure 10C**) mannose inhibition reduced viability about 2-logs in comparison to control groups for both phagocytes. Similar behavior was observed for mannose inhibition during *Ca* and *Hc* interactions with RAW macrophages. However, for *Cn*, despite the lower absolute CFU numbers in the presence of mannose, the normalization by the symmetrized internalization index (**Figure 10B**) demonstrated overall similar viability upon interactions with amoeba (**Figure 10B**), whereas a reduction was observed for *Cn* interaction with macrophages.

### Proteomic Characterization of Phagocytes’ MPPs

**Figure 11:**
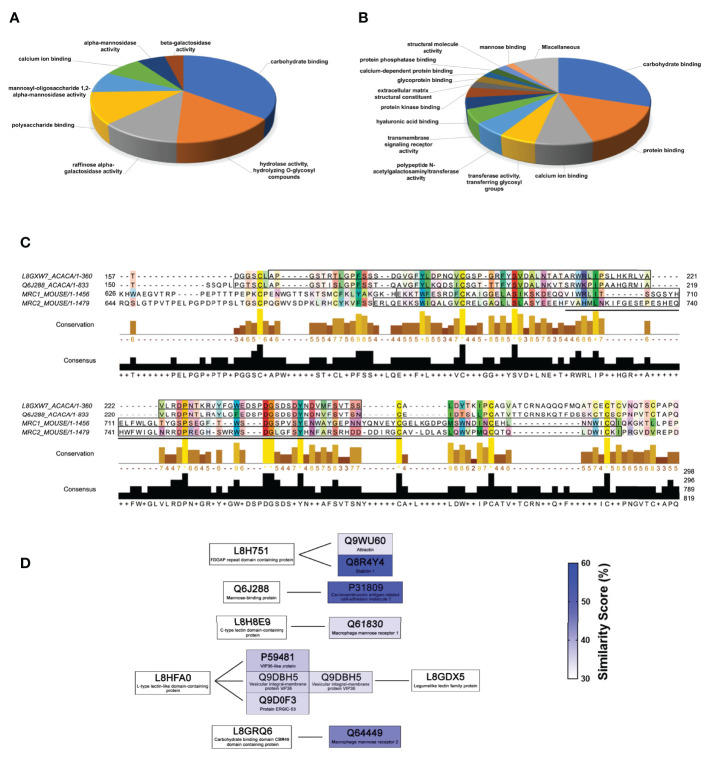
Mannose-binding proteins from *Acanthamoeba castellanii* and RAW macrophages exhibited identity in domains with lectin properties. Proteins identified in MPPs of **(A)** *A. castellanii (Ac)* and **(B)** RAW 246.7 macrophages were annotated using the DAVID annotation tools and classified according to their molecular functions. For both MPPs pools, carbohydrate binding proteins comprised the main category, **(C)** *In silico* alignment of the *Ac* transmembrane proteins MBP (L8GXW7) and MBP1 (Q6J288) with the RAW receptors Mrc1 (Q61830) and Mrc2 (Q64449), using the multiple sequence alignment algorithm, Clustal Omega by Jalview Software. Amino acids on colored background show highly conserved residues. The DUF4114 domains of *Ac* proteins and C-type lectin-like domains of RAW receptors are highlighted by a black rectangle, while the domains equivalent to the secondary structure of ConA-like lectins/glucanases superfamily are indicated by black underlining. **(D)** From the MPPs identified in *Ac*, six displayed high similarity to proteins also found in the macrophage MBPs pools, suggesting molecular mimicry.

After the isolation of MPPs of *Ac* and RAW, the samples were processed for identification by mass spectrometry. In the *Ac* MPPs pool, 43 proteins were identified and relatively quantified (**Supplementary Table 1**), whose molecular function annotation classified the vast majority of proteins as carbohydrate binding (GO:0030246) and seven other main groups (**Figure 11A**). For macrophages instead, 120 MPPs were identified (**Supplementary Table 2**) and fell into 15 distinct molecular functions (**Figure 11B**), with the vast majority demonstrating the carbohydrate binding function (GO:0030246).

Regarding specifically the literature recognized mannose binding lectins of *Ac* (Gonçalves et al., 2019), the transmembrane protein MBP1 (Q6J288) was the most abundant (2.6%), followed by the MBP (L8GXW7, 0.7%). In RAW macrophages, the mannose receptor Mrc2 (Q64449) was the most abundant (0.8%), followed by the Mrc1 (Q61830; 0.7%), both featuring transmembrane helical domain. Alignment of the sequence of these knows mannose receptors from both organisms demonstrated an overlapping of the DUF4114 domains of *Ac* proteins and the C-type lectin-like domains of RAW receptors and denoted a perfect alignment of the regions equivalent to the secondary structure of ConA-like lectins/glucanases superfamily in all proteins (**Figure 11C**).

Protein sequences of identified *Ac* MPPs (**Supplementary Table 1**) were used as query to find the highest similarity among macrophage identified MPPs (**Supplementary Table 2**), and in this subset specifically, six amoeba proteins, including the MBPs Q6J288 and five other lectins, had mimetic macrophages proteins with high similarity (**Figure 11D**). Overall, amoebas seem to possess a simpler repertory for mannose binding and lower number of analog genes, whereas MPPs from macrophages represented a more diverse group, which could be the result of paralogical genetic evolution from ancestor genes.

## Discussion

*Acanthamoeba*, a free lifestyle and ubiquitous protozoan, has been standing out as an important host for a myriad of microorganisms in the environment (Guimaraes et al., 2016), which favors a wide range of opportunities for interaction to distinct individuals and the generation of a complex network (Casadevall et al., 2003; Rizzo et al., 2017; da Silva Ferreira et al., 2021). Several authors raise the hypothesis that the emergence of mammalian-fungal virulence could be the result of pressures established by soil predators, as *A. castellanii*, which acting as a “Trojan horse” for fungi, may select their virulence traits, enabling them to establish infections in more evolutionarily complex hosts (Steenbergen and Casadevall, 2003; Guimaraes et al., 2016; da Silva Ferreira et al., 2021). In fact, several studies demonstrate that the interaction between *Ac* and fungi enhances fungal resistance strategies within these soil predators, which are important for survival and fungal pathogenicity in animal models, from as simple invertebrate model, the lepidoptera larvae *Galleria mellonella*, to murine models (Steenbergen et al., 2001; Chaffin, 2008; Kruppa, 2009; Chrisman et al., 2011; Gow et al., 2017; Latge et al., 2017; Wang, 2018; Casadevall et al., 2019a; Cortes et al., 2019; Gonçalves et al., 2019; Zaragoza, 2019; Maliehe et al., 2020).

The fungal cell wall, the main target for phagocyte recognition (Alcazar-Fuoli et al., 2020), is mostly composed by polysaccharide fibers and several anchored proteins responsible for promoting vital functions to fungi (Gow et al., 2017; Latge et al., 2017; Cortes et al., 2019). Among fungal species, the cell wall has a widely variable organization (Garcia-Rubio et al., 2019), which justifies the approach of three different fungi evaluated in the present work. *Ca* has the outer layer of the cell wall rich in mannoproteins, which consist of proteins associated with O- or N-linked mannans (Chaffin, 2008; Kruppa, 2009). In *C. neoformans*, anchored to the cell wall is the cryptococcal capsule, which consists of glucuronoxylomannan (GXM) and glururonoxylomanogalactan (GXMGal) polymers, along with some mannoproteins (Gow et al., 2017; Wang, 2018). *Hc* has a glucan-rich cell wall where β-1,3-glucan is a pathogen-associate molecular pattern (PAMP)that modulates the host’s immune response system, whereas the external α-1-3-glucan plays a major role in the immune escape and fungal virulence (Guimarães et al., 2011a).

The prevalence of mannoproteins on the fungal surface highlight the importance of mannans among the polysaccharides involved in fungal pathogenesis. To immunological recognition, the Mannose Receptor (MR) and MBL- Pathogen Recognition Receptors (PRRs) of the C-tyle lectin receptors (CLRs) family- are active participants in the interaction between fungi and macrophages (Brown et al., 2018; Kirkland and Fierer, 2020; Nikolakopoulou et al., 2020). In the same way, our group identified two mannose recognizing transmembrane proteins on the ameboid surface, a Mannose-Binding Protein (MBP, L8GXW7) and a Mannose-Binding Protein-1 (MBP1, Q6J288), as the main receptors for universal fungal recognition, granting access to the amoeboid intracellular environment, which could affect the fungal virulence and potentialize the impact of these pathogens on superior hosts (Casadevall et al., 2019b; Gonçalves et al., 2019).

(Siddiqui and Khan 2012a, 2012b) hypothesize that *Acanthamoeba* is a possible common ancestor of macrophages, as these phagocytes, besides sharing similarities on cell morphology and ultrastructure, biology, and phagocytosis, also display conserved pathogen´s recognition mechanisms. The MBLs identified in fungal recognition by both amoeboid and macrophage surfaces, belong to a ConA-like super family proteins, suggesting the possibility of convergent evolution (Gonçalves et al., 2019; Nikolakopoulou et al., 2020). However, despite evidence, the relative importance of MBLs in the interaction of each phagocyte has not been determined and explored in the literature to corroborate this hypothesis. In the present study, we seek to explore the mechanisms of interaction between amoeba and macrophages with pathogenic fungi, molecularly investigating the profiles and characterizing the activity of mannose recognizing proteins to fungal cell wall mannoproteins and mannans, and possibly establish degrees of similarity between these organisms. Affinity chromatography initially suggested that differences in elution profiles of proteins with affinity to mannose present on the phagocytes’ surface reflects distinct affinity of MPPs, as lower mannose concentrations were sufficient for detachment of amoeboid proteins, as opposed to higher mannose-concentrations of macrophages MPPs, indicating the presence of greater affinity by macrophages.

A MBP from *Ac* characterized in previous studies revealed a 400 KDa protein, with 130 KDa subunits composed of a large extracellular N-terminal domain, with a region rich in cysteine; a transmembrane domain, and a small C-terminal cytoplasmic domain (Garate et al., 2004; Garate et al., 2006). Mammalian immune cells, on the other hand, express MBPs ranging from 200 KDa to 650 KDa, with 31 KDa subunits forming a trimer hexamer, with 18 identical subunits (Sheriff et al., 1994; Weis and Drickamer, 1994; Fraser et al., 1998). Each subunit has four distinct domains: a cysteine-rich domain, a collagen-like domain, a coil region and a calcium-dependent domain (Sheriff et al., 1994; Weis and Drickamer, 1994). The cluster of three calcium-dependent domains by elongated and branched oligosaccharides, such as those found in yeast cell walls and other microorganisms, allows receptors to establish high-affinity bonds and display themselves as ideally configured, arranged for strong interactions between MBPs of superior hosts and pathogens (Weis and Drickamer, 1994; Fraser et al., 1998).

The identification of bands of approximately 55 KDa, 75 KDa, 110 KDa and 150 KDa by Western blots in RAW macrophages corroborates with existing descriptions of the greater structural complexity of the receptor of macrophage compared to the amoeba. However, the enrichment process allowed us to confirm the presence of a ~130 KDa, a *Ac* MBP1- (Garate et al., 2004) and a 55 KDa MBP described by our group as proteins with high affinity to yeasts (Gonçalves et al., 2019). In macrophages, low molecular weight protein subunits have been identified and probably correspond to the 31 KDa subunits, described in previous studies (Sheriff et al., 1994; Weis and Drickamer, 1994; Fraser et al., 1998). The mannose receptor CD206 was previously identified by Western blot assays, with bands of approximately 170 and 175 KDa corresponding to soluble and transmembrane forms (Wileman et al., 1986; Rødgaard-Hansen et al., 2014). This information corroborates the presence of the band corresponding to >150 KDa, identified upon the purification process.

The success of enrichment process in MPPs purification is demonstrated by the more intense binding to targets on the fungal cell wall in comparison to total extracts. Regarding the quantitative differences between the phagocytes, macrophage MPPs bound more intensely to the yeasts compared to the ameboids, by both ELISA and flow cytometry, which could be explained by the lower heterogeneity of surface proteins recognizing the fungal cell wall of the last. However, qualitative binding evaluation of MPPs from both models provides a point-less binding pattern, which co-localized with the mannose-affinity lectin ConA and suggested an overlapping recognition on each cell wall model. In addition, regardless of the fungal model, MPPs of *Ac* and RAW targeted similar fungal cell wall targets, and cross-inhibited each other in the interaction assays. This could be translated also in terms of evolutionarily developed fungal escaping mechanisms, since masking or reduced expression of ligands on fungal cell wall to avoid recognition and predation by amoeba in the environment could result in the same phenomena toward macrophages *in vivo* (Casadevall et al., 2003; Gonçalves et al., 2019; Maliehe et al., 2020).

The inhibition with soluble mannose impacted all the interaction processes evaluated. The highest percentages of inhibition occurred in the interaction between fungi and *Ac*, justifying the MBPs as main route of fungal interaction in amoebae (Gonçalves et al., 2019). The macrophages display a higher binding capacity and consequently, a wider ability to recognize the most diverse polysaccharides present on the fungal cell wall, potentiating the fungal recognition by these phagocytes and triggering a broader and more complex antifungal response. With inhibition detected within 30 min of interaction, mannose recognition seems to be an immediate route for fungal adherence and internalization, regardless of the phagocyte. In *Ac*, the late interaction seems to be more impacted, whereas in macrophages, other existing routes may compensate for the interruption of this pathway, such as the Dectin-1 receptor, specific for the recognition of fungal β-glucans (Goyal et al., 2018).

The blockade of the mannose receptor directly affected fungal viability inside phagocytes. By monitoring antifungal activity with a fungal viability probe, we observed that the mannose recognition plays a role on the fungal survival inside the amoeba as the interaction *via* MBPs might result in the adaptation of pathogens inside these protozoa. However, additional experiments need to be carried out to understand, molecularly, the fate of yeasts inside amoeba after phagocytosis. Along in macrophages, there were higher percentages of non-viable yeasts in the groups inhibited with mannose. Therefore, interactions occurring through pathways other than mannose recognition might activate fungicidal activity of phagocytes.

Finally, the protein characterization within the MPP’s pools confirmed the presence of the amoeboid receptors MBP (L8GXW7) and MBP1 (Q6J288), as well as the lectins Mrc1- Macrophage mannose receptor 1 (Q61830), and Mrc2- C-type mannose receptor 2 (Q64449), in addition to the participation of other proteins with transmembrane domains in the recognition of mannose in both models. The alignment between these proteins displayed conserved regions in the carbohydrate binding domains, mainly between the transmembrane proteins MBP1, Mrc1 and Mrc2. It is possible to justify the complexity of RAW macrophage receptors, as already observed in results throughout this work, by the presence of eight Lectin C-type domains in each of the structures of Mrc1 and Mrc2 (Q61830 and Q64449), compared to a short Con-A like domain in MBP and MBP-1 (L8GXW7 and Q6J288). However, the abundant presence of highly conserved residues among these proteins leads to the proposal of evolution of this interaction pathway through the recognition of mannosylated residues. Probably, in simpler organisms, such as amoeba, the interaction *via* MBPs is widely used for nutritional purposes and for the survival of amoeboid organisms. Throughout evolution these pathways were not lost; they are complex in higher organisms, as in immune cells, such as macrophages, a cell model used for comparison in this work.

Besides fungi, other pathogens also recognized by mannose receptors of immune cells can be found inside amoeboid cells. This is the case of Coronaviruses, such as SARS-CoV-1 and SARS-CoV-2, the causative agents of the pandemic Severe Acute Respiratory Syndrome (SARS) in humans, currently considered by the World Health Organization (WHO) as a global public health emergency (Madhav et al., 2017; Chatterjee et al., 2020; Yang et al., 2020). We know that mannose receptors are key molecules in the innate immune responses against these viruses and that, possibly, *Acanthamoeba* can harbor and supply new coronaviruses to the environment, contributing to the infection of susceptible hosts (Zhang et al., 2005; Tu et al., 2015; Siddiqui and Ahmed Khan, 2020; Siddiqui and Khan, 2020b; Siddiqui and Khan, 2020a; Ip et al., 2005). Likewise, resistant bacteria such as *Streptococcus pneumoniae*, interact with *Ac*, selecting traces of pathogenicity within this host (Evstigneeva et al., 2009; Macedo-Ramos et al., 2011; Macedo-Ramos et al., 2014; Denet et al., 2017; Siddiqui et al., 2017). *Acanthamoeba* being a major environmental reservoir for potential pathogens contributes to the emergence of epidemics (Greub and Raoult, 2004; Liu et al., 2019).

Due to the fundamental role of MBLs in the specificity and the high affinity recognition of universal antigens by phagocytic cells, there is a need to expand the knowledge, with comparative studies of how these lectins recognize microorganisms, specially pathogenic fungus, in order to provide subsidies to reduce the pathogen’s impact on subsequent mammalian infections and promote approaches for future therapeutic interventions and mycological diagnosis, the main challenges in the current scenario.

## Data Availability Statement

The datasets presented in this study can be found in online repositories. The link to the repository and accession number can be found below: http://www.ebi.ac.uk/pride; PXD031121.

## Author Contributions

All authors contributed to conception and design of the study. MF, SM, DG, CR, LH, LR, and AG performed the experiments. MF, SM, DG, and AG organized the database. MF, SM, DG, and AG performed the statistical analysis. All authors designed experiments and participated in scientific discussions. MF, SM, LN, and AG wrote the first draft of the manuscript. All authors contributed to the article and approved the submitted version.

## Funding

AG was supported by grants from the Brazilian agencies Conselho Nacional de Desenvolvimento Científico e Tecnológico (CNPq, grant 311470/2018-1) and Fundação Carlos Chagas de Amparo à Pesquisa no Estado do Rio de Janeiro (FAPERJ E-26/202.696/2018).

## Conflict of Interest

The authors declare that the research was conducted in the absence of any commercial or financial relationships that could be construed as a potential conflict of interest.

## Publisher’s Note

All claims expressed in this article are solely those of the authors and do not necessarily represent those of their affiliated organizations, or those of the publisher, the editors and the reviewers. Any product that may be evaluated in this article, or claim that may be made by its manufacturer, is not guaranteed or endorsed by the publisher.

## Supplementary Material

Supplementary Table 1 Listing of proteins identified by mass spectrometry and *de novo* sequencing analysis in *Acanthamoeba castellanii* mannose purified proteins (MPPs). Experimental and biological duplicates were performed, and spectra counts were validated by *De novo* sequencing analysis. MPPs’ candidates and their UniprotKB entries are indicated in the table. Likewise, proteins that have a transmembrane helix domain are flagged (underline). The percentages of protein abundance in the samples were determined as follows: SpecCounts of protein/∑SpecCounts of all proteins validated x 100. The absolute average and percentages of protein abundance among the samples are displayed in the table. All identified proteins are part of the database of lectins of the species *Acanthamoeba castellanii*, obtained from https://www.uniprot.org/.

Supplementary Table 2 Listing of lectins validated by *De novo* sequencing analysis in RAW mannose purified proteins (MPPs). Other carbohydrate-affinity lectins were identified in the samples, demonstrating cross-linking to mannose. Likewise, proteins that have a transmembrane helix domain are pointed (underline) and their absolute averages and percentages of abundance within samples are displayed in the table. All identified proteins are part of the database reviewed of lectins of the species *Mus musculus*, obtained from https://www.uniprot.org/.
