# Supplementary material for: Recognition of Cell Wall Mannosylated Components as a Conserved Feature for Fungal Entrance, Adaptation and Survival Within Trophozoites of Acanthamoeba castellanii and Murine Macrophages
Source: Front Cell Infect Microbiol. 2022 May 31;12:858979. doi: 10.3389/fcimb.2022.858979 (PMC9194641; doi:10.3389/fcimb.2022.858979)
Supplement: Supplementary Table 1 — Listing of proteins identified by mass spectrometry and de novo sequencing analysis in Acanthamoeba castellanii mannose purified proteins (MPPs). Experimental and biological duplicates were performed, and spectra counts were validated by De novo sequencing analysis. MPPs’ candidates and their UniprotKB entries are indicated in the table. Likewise, proteins that have a transmembrane helix domain are flagged (underline). The percentages of protein abundance in the samples were determined as follows: SpecCounts of protein/∑SpecCounts of all proteins validated x 100. The absolute average and percentages of protein abundance among the samples are displayed in the table. All identified proteins are part of the database of lectins of the species Acanthamoeba castellanii, obtained from https://www.uniprot.org/. [file Table_1.docx]

|  |  | Samples | | | | | | | | | | |  | | | |  | |  |
| --- | --- | --- | --- | --- | --- | --- | --- | --- | --- | --- | --- | --- | --- | --- | --- | --- | --- | --- | --- |
|  |  | 1 | | 2 | | | 3 | | | 4 | | | |  | | | |  |  |
| **Uniprot ID** | **Protein Name** | **SpecCounts** | **Abundance** | | **SpecCounts** | **Abundance** | | **SpecCounts** | **Abundance** | | **SpecCounts** | **Abundance** | | | **Average abundance** | **% Abundance** | | | |
| L8HAB9 | BTB/POZ domain containing protein | 22 | 0,0692 | | 27 | 0,0748 | | 9 | 0,0288 | | 21 | 0,0766 | | | 0,0624 | 6,24 | | | |
| L8GUS2 | FGGAP repeat domain containing protein | 27 | 0,0849 | | 22 | 0,0609 | | 13 | 0,0417 | | 12 | 0,0438 | | | 0,0578 | 5,78 | | | |
| L8HEV2 | Alpha-mannosidase | 13 | 0,0409 | | 20 | 0,0554 | | 20 | 0,0641 | | 11 | 0,0401 | | | 0,0501 | 5,01 | | | |
| L8H070 | Alpha-mannosidase | 12 | 0,0377 | | 21 | 0,0582 | | 17 | 0,0545 | | 9 | 0,0328 | | | 0,0458 | 4,58 | | | |
| L8GX61 | Alpha-mannosidase | 15 | 0,0472 | | 14 | 0,0388 | | 9 | 0,0288 | | 16 | 0,0584 | | | 0,0433 | 4,33 | | | |
| L8GX06 | Beta-mannosidase | 12 | 0,0377 | | 13 | 0,0360 | | 13 | 0,0417 | | 13 | 0,0474 | | | 0,0407 | 4,07 | | | |
| L8GG61 | EGFlike domain containing protein | 11 | 0,0346 | | 25 | 0,0693 | | 10 | 0,0321 | | 6 | 0,0219 | | | 0,0394 | 3,94 | | | |
| L8HAA0 | alpha-1,2-Mannosidase | 18 | 0,0566 | | 9 | 0,0249 | | 9 | 0,0288 | | 9 | 0,0328 | | | 0,0358 | 3,58 | | | |
| L8H5J8 | Beta-galactosidase | 9 | 0,0283 | | 16 | 0,0443 | | 11 | 0,0353 | | 9 | 0,0328 | | | 0,0352 | 3,52 | | | |
| L8GYR5 | Legume lectins beta domain containing protein | 9 | 0,0283 | | 11 | 0,0305 | | 13 | 0,0417 | | 11 | 0,0401 | | | 0,0351 | 3,51 | | | |
| L8GR22 | alpha-1,2-Mannosidase | 8 | 0,0252 | | 14 | 0,0388 | | 17 | 0,0545 | | 5 | 0,0182 | | | 0,0342 | 3,42 | | | |
| L8HDD6 | Filamin repeat domain containing protein | 16 | 0,0503 | | 10 | 0,0277 | | 7 | 0,0224 | | 9 | 0,0328 | | | 0,0333 | 3,33 | | | |
| L8H751 | FGGAP repeat domain containing protein | 10 | 0,0314 | | 7 | 0,0194 | | 8 | 0,0256 | | 13 | 0,0474 | | | 0,0310 | 3,10 | | | |
| L8GZ33 | alpha-1,2-Mannosidase | 9 | 0,0283 | | 9 | 0,0249 | | 10 | 0,0321 | | 10 | 0,0365 | | | 0,0304 | 3,04 | | | |
| L8H1N2 | Filamin repeat domain containing protein | 9 | 0,0283 | | 9 | 0,0249 | | 8 | 0,0256 | | 11 | 0,0401 | | | 0,0298 | 2,98 | | | |
| L8GT56 | FGGAP repeat domain containing protein | 5 | 0,0157 | | 8 | 0,0222 | | 10 | 0,0321 | | 10 | 0,0365 | | | 0,0266 | 2,66 | | | |
| Q6J288 | Mannose-binding protein | 7 | 0,0220 | | 15 | 0,0416 | | 7 | 0,0224 | | 5 | 0,0182 | | | 0,0261 | 2,61 | | | |
| L8HCH4 | Beta-mannosidase | 9 | 0,0283 | | 10 | 0,0277 | | 5 | 0,0160 | | 8 | 0,0292 | | | 0,0253 | 2,53 | | | |
| L8H589 | alpha-1,2-Mannosidase | 6 | 0,0189 | | 6 | 0,0166 | | 9 | 0,0288 | | 9 | 0,0328 | | | 0,0243 | 2,43 | | | |
| L8GFS3 | Alpha-galactosidase | 7 | 0,0220 | | 5 | 0,0139 | | 11 | 0,0353 | | 6 | 0,0219 | | | 0,0233 | 2,33 | | | |
| L8GDX5 | Legumelike lectin family protein | 8 | 0,0252 | | 3 | 0,0083 | | 10 | 0,0321 | | 4 | 0,0146 | | | 0,0200 | 2,00 | | | |
| L8H838 | Beta-galactosidase | 4 | 0,0126 | | 11 | 0,0305 | | 7 | 0,0224 | | 3 | 0,0109 | | | 0,0191 | 1,91 | | | |
| L8H8E9 | C-type lectin domain-containing protein | 7 | 0,0220 | | 4 | 0,0111 | | 3 | 0,0096 | | 7 | 0,0255 | | | 0,0171 | 1,71 | | | |
| L8HHU7 | Alpha-galactosidase | 6 | 0,0189 | | 7 | 0,0194 | | 4 | 0,0128 | | 4 | 0,0146 | | | 0,0164 | 1,64 | | | |
| L8HFA0 | L-type lectin-like domain-containing protein | 4 | 0,0126 | | 5 | 0,0139 | | 4 | 0,0128 | | 5 | 0,0182 | | | 0,0144 | 1,44 | | | |
| L8H4V5 | Rhamnosebinding lectin (SAL), putative | 5 | 0,0157 | | 4 | 0,0111 | | 5 | 0,0160 | | 4 | 0,0146 | | | 0,0144 | 1,44 | | | |
| L8GN31 | Alpha-galactosidase | 3 | 0,0094 | | 2 | 0,0055 | | 6 | 0,0192 | | 6 | 0,0219 | | | 0,0140 | 1,40 | | | |
| L8GP58 | Alphamannosidase | 1 | 0,0031 | | 9 | 0,0249 | | 5 | 0,0160 | | 3 | 0,0109 | | | 0,0138 | 1,38 | | | |
| L8GMY9 | Alpha-galactosidase | 3 | 0,0094 | | 4 | 0,0111 | | 6 | 0,0192 | | 4 | 0,0146 | | | 0,0136 | 1,36 | | | |
| L8GWP4 | Carbohydrate binding domain cbm49 protein | 3 | 0,0094 | | 2 | 0,0055 | | 7 | 0,0224 | | 4 | 0,0146 | | | 0,0130 | 1,30 | | | |
| L8GSR8 | Htype lectin domain containing protein | 5 | 0,0157 | | 7 | 0,0194 | | 4 | 0,0128 | | 1 | 0,0036 | | | 0,0129 | 1,29 | | | |
| L8GRK2 | Mannose-P-dolichol utilization defect 1 protein homolog | 5 | 0,0157 | | 3 | 0,0083 | | 4 | 0,0128 | | 4 | 0,0146 | | | 0,0129 | 1,29 | | | |
| L8HAP9 | Carbohydrate binding domain CBM49 domain containing protein | 3 | 0,0094 | | 5 | 0,0139 | | 4 | 0,0128 | | 3 | 0,0109 | | | 0,0118 | 1,18 | | | |
| L8GV32 | Carbohydrate binding domain cbm49 protein | 4 | 0,0126 | | 3 | 0,0083 | | 4 | 0,0128 | | 2 | 0,0073 | | | 0,0103 | 1,03 | | | |
| L8GWL0 | Alpha-galactosidase | 6 | 0,0189 | | 2 | 0,0055 | | 4 | 0,0128 | | 1 | 0,0036 | | | 0,0102 | 1,02 | | | |
| L8HAP7 | Galactose binding lectin domain containing protein | 3 | 0,0094 | | 3 | 0,0083 | | 1 | 0,0032 | | 4 | 0,0146 | | | 0,0089 | 0,89 | | | |
| L8H444 | SUEL-type lectin domain-containing protein | 4 | 0,0126 | | 2 | 0,0055 | | 4 | 0,0128 | | 1 | 0,0036 | | | 0,0086 | 0,86 | | | |
| L8GRQ6 | Carbohydrate binding domain CBM49 domain containing protein | 3 | 0,0094 | | 1 | 0,0028 | | 2 | 0,0064 | | 3 | 0,0109 | | | 0,0074 | 0,74 | | | |
| L8GXW7 | Mannosebinding protein | 2 | 0,0063 | | 2 | 0,0055 | | 5 | 0,0160 | | 0 | 0,0000 | | | 0,0070 | 0,70 | | | |
| L8H247 | Carbohydrate binding domain cbm49 protein | 2 | 0,0063 | | 6 | 0,0166 | | 0 | 0,0000 | | 1 | 0,0036 | | | 0,0066 | 0,66 | | | |
| L8GT02 | Carbohydrate binding domain cbm49 protein | 1 | 0,0031 | | 2 | 0,0055 | | 3 | 0,0096 | | 2 | 0,0073 | | | 0,0064 | 0,64 | | | |
| L8HE10 | Alpha-galactosidase | 1 | 0,0031 | | 1 | 0,0028 | | 1 | 0,0032 | | 4 | 0,0146 | | | 0,0059 | 0,59 | | | |
| L8GSU2 | Carbohydrate binding domain cbm49 protein | 1 | 0,0031 | | 2 | 0,0055 | | 3 | 0,0096 | | 1 | 0,0036 | | | 0,0055 | 0,55 | | | |
|  | Total | 318 |  | | 361 |  | | 312 |  | | 274 |  | | | 1 | 100 | | | |
