# Supplementary material for: Recognition of Cell Wall Mannosylated Components as a Conserved Feature for Fungal Entrance, Adaptation and Survival Within Trophozoites of Acanthamoeba castellanii and Murine Macrophages
Source: Front Cell Infect Microbiol. 2022 May 31;12:858979. doi: 10.3389/fcimb.2022.858979 (PMC9194641; doi:10.3389/fcimb.2022.858979)
Supplement: Supplementary Table 2 — Listing of lectins validated by De novo sequencing analysis in RAW mannose purified proteins (MPPs). Other carbohydrate-affinity lectins were identified in the samples, demonstrating cross-linking to mannose. Likewise, proteins that have a transmembrane helix domain are pointed (underline) and their absolute averages and percentages of abundance within samples are displayed in the table. All identified proteins are part of the database reviewed of lectins of the species Mus musculus, obtained from https://www.uniprot.org/. [file Table_2.docx]

|  |  | Samples | | | | | | | |  |  |
| --- | --- | --- | --- | --- | --- | --- | --- | --- | --- | --- | --- |
|  |  | 1 | | 2 | | 3 | | 4 | |  |  |
| **Uniprot ID** | **Protein Names** | **SpecCounts** | **Abundance** | **SpecCounts** | **Abundance** | **SpecCounts** | **Abundance** | **SpecCounts** | **Abundance** | **Average abundance** | **% Abundance** |
| Q9QYX7 | Protein piccolo | 15 | 0,0490 | 10 | 0,0350 | 16 | 0,0510 | 15 | 0,0530 | 0,0470 | 4,70 |
| O88737 | Protein bassoon | 12 | 0,0392 | 13 | 0,0455 | 8 | 0,0255 | 10 | 0,0353 | 0,0364 | 3,64 |
| Q62059 | Versican core protein | 9 | 0,0294 | 9 | 0,0315 | 6 | 0,0191 | 4 | 0,0141 | 0,0235 | 2,35 |
| Q62261 | Spectrin beta chain, non-erythrocytic 1 | 8 | 0,0261 | 8 | 0,0280 | 5 | 0,0159 | 5 | 0,0177 | 0,0219 | 2,19 |
| Q7TN88 | Polycystic kidney disease protein 1-like 2 | 13 | 0,0425 | 5 | 0,0175 | 6 | 0,0191 | 2 | 0,0071 | 0,0215 | 2,15 |
| Q8C102 | Polypeptide N-acetylgalactosaminyltransferase 5 | 6 | 0,0196 | 1 | 0,0035 | 9 | 0,0287 | 8 | 0,0283 | 0,0200 | 2,00 |
| O08852 | Polycystin-1 | 6 | 0,0196 | 4 | 0,0140 | 10 | 0,0318 | 4 | 0,0141 | 0,0199 | 1,99 |
| Q80TS3 | Adhesion G protein-coupled receptor L3 | 7 | 0,0229 | 8 | 0,0280 | 5 | 0,0159 | 4 | 0,0141 | 0,0202 | 2,02 |
| Q8R4Y4 | Stabilin-1 | 5 | 0,0163 | 8 | 0,0280 | 3 | 0,0096 | 7 | 0,0247 | 0,0197 | 1,97 |
| Q6GQT1 | Alpha-2-macroglobulin-P | 3 | 0,0098 | 3 | 0,0105 | 11 | 0,0350 | 5 | 0,0177 | 0,0182 | 1,82 |
| Q8R4U0 | Stabilin-2 | 9 | 0,0294 | 4 | 0,0140 | 4 | 0,0127 | 5 | 0,0177 | 0,0185 | 1,85 |
| A2AQ25 | Sickle tail protein | 5 | 0,0163 | 5 | 0,0175 | 3 | 0,0096 | 8 | 0,0283 | 0,0179 | 1,79 |
| Q60767 | Lymphocyte antigen 75 | 4 | 0,0131 | 5 | 0,0175 | 6 | 0,0191 | 5 | 0,0177 | 0,0168 | 1,68 |
| P04104 | Keratin, type II cytoskeletal 1 | 7 | 0,0229 | 2 | 0,0070 | 7 | 0,0223 | 3 | 0,0106 | 0,0157 | 1,57 |
| Q61543 | Golgi apparatus protein 1 | 4 | 0,0131 | 4 | 0,0140 | 3 | 0,0096 | 7 | 0,0247 | 0,0153 | 1,53 |
| P01029 | Complement C4-B | 2 | 0,0065 | 5 | 0,0175 | 8 | 0,0255 | 3 | 0,0106 | 0,0150 | 1,50 |
| Q3UWA6 | Heat-stable enterotoxin receptor | 3 | 0,0098 | 6 | 0,0210 | 6 | 0,0191 | 3 | 0,0106 | 0,0151 | 1,51 |
| Q80Z38 | SH3 and multiple ankyrin repeat domains protein 2 | 7 | 0,0229 | 6 | 0,0210 | 0 | 0,0000 | 5 | 0,0177 | 0,0154 | 1,54 |
| Q80TR1 | Adhesion G protein-coupled receptor L1 | 2 | 0,0065 | 7 | 0,0245 | 4 | 0,0127 | 4 | 0,0141 | 0,0145 | 1,45 |
| O35927 | Catenin delta-2 | 8 | 0,0261 | 2 | 0,0070 | 3 | 0,0096 | 4 | 0,0141 | 0,0142 | 1,42 |
| Q9WU60 | Attractin | 3 | 0,0098 | 4 | 0,0140 | 5 | 0,0159 | 4 | 0,0141 | 0,0135 | 1,35 |
| P35329 | B-cell receptor CD22 | 4 | 0,0131 | 6 | 0,0210 | 1 | 0,0032 | 3 | 0,0106 | 0,0120 | 1,20 |
| Q8JZZ7 | Adhesion G protein-coupled receptor L2 | 7 | 0,0229 | 1 | 0,0035 | 2 | 0,0064 | 4 | 0,0141 | 0,0117 | 1,17 |
| Q684R7 | FRAS1-related extracellular matrix protein 1 | 2 | 0,0065 | 3 | 0,0105 | 3 | 0,0096 | 5 | 0,0177 | 0,0111 | 1,11 |
| P08553 | Neurofilament medium polypeptide | 4 | 0,0131 | 1 | 0,0035 | 2 | 0,0064 | 6 | 0,0212 | 0,0110 | 1,10 |
| Q2EG98 | Polycystic kidney disease protein 1-like 3 | 2 | 0,0065 | 4 | 0,0140 | 3 | 0,0096 | 4 | 0,0141 | 0,0111 | 1,11 |
| Q8K2C7 | Protein OS-9 | 2 | 0,0065 | 4 | 0,0140 | 4 | 0,0127 | 3 | 0,0106 | 0,0110 | 1,10 |
| Q8CC35 | Synaptopodin | 3 | 0,0098 | 3 | 0,0105 | 4 | 0,0127 | 3 | 0,0106 | 0,0109 | 1,09 |
| Q8K4Q8 | Collectin-12 | 6 | 0,0196 | 3 | 0,0105 | 3 | 0,0096 | 1 | 0,0035 | 0,0108 | 1,08 |
| Q62417 | Sorbin and SH3 domain-containing protein 1 | 2 | 0,0065 | 1 | 0,0035 | 5 | 0,0159 | 4 | 0,0141 | 0,0100 | 1,00 |
| Q8K297 | Procollagen galactosyltransferase 1 | 3 | 0,0098 | 3 | 0,0105 | 4 | 0,0127 | 2 | 0,0071 | 0,0100 | 1,00 |
| P35564 | Calnexin | 1 | 0,0033 | 3 | 0,0105 | 3 | 0,0096 | 4 | 0,0141 | 0,0094 | 0,94 |
| P29351 | Tyrosine-protein phosphatase non-receptor type 6 | 3 | 0,0098 | 1 | 0,0035 | 4 | 0,0127 | 3 | 0,0106 | 0,0092 | 0,92 |
| Q91WP0 | Mannan-binding lectin serine protease 2 | 3 | 0,0098 | 4 | 0,0140 | 4 | 0,0127 | 0 | 0,0000 | 0,0091 | 0,91 |
| P55066 | Neurocan core protein | 1 | 0,0033 | 1 | 0,0035 | 4 | 0,0127 | 4 | 0,0141 | 0,0084 | 0,84 |
| Q8BMB0 | BRCA2-interacting transcriptional repressor EMSY | 0 | 0,0000 | 3 | 0,0105 | 4 | 0,0127 | 3 | 0,0106 | 0,0085 | 0,85 |
| Q6A051 | Attractin-like protein 1 | 2 | 0,0065 | 2 | 0,0070 | 2 | 0,0064 | 4 | 0,0141 | 0,0085 | 0,85 |
| P59759 | Myocardin-related transcription factor B | 2 | 0,0065 | 1 | 0,0035 | 4 | 0,0127 | 3 | 0,0106 | 0,0083 | 0,83 |
| P98154 | Integral membrane protein DGCR2/IDD | 2 | 0,0065 | 2 | 0,0070 | 3 | 0,0096 | 3 | 0,0106 | 0,0084 | 0,84 |
| Q62028 | Secretory phospholipase A2 receptor | 1 | 0,0033 | 3 | 0,0105 | 6 | 0,0191 | 0 | 0,0000 | 0,0082 | 0,82 |
| Q64449 | C-type mannose receptor 2 | 2 | 0,0065 | 4 | 0,0140 | 3 | 0,0096 | 1 | 0,0035 | 0,0084 | 0,84 |
| Q80VP0 | Tectonin beta-propeller repeat-containing protein 1 | 5 | 0,0163 | 1 | 0,0035 | 2 | 0,0064 | 2 | 0,0071 | 0,0083 | 0,83 |
| O35206 | Collagen alpha-1(XV) chain | 1 | 0,0033 | 1 | 0,0035 | 5 | 0,0159 | 2 | 0,0071 | 0,0074 | 0,74 |
| O88935 | Synapsin-1 | 2 | 0,0065 | 3 | 0,0105 | 0 | 0,0000 | 4 | 0,0141 | 0,0078 | 0,78 |
| Q61830 | Macrophage mannose receptor 1 | 3 | 0,0098 | 0 | 0,0000 | 4 | 0,0127 | 2 | 0,0071 | 0,0074 | 0,74 |
| Q01102 | P-selectin | 1 | 0,0033 | 4 | 0,0140 | 4 | 0,0127 | 0 | 0,0000 | 0,0075 | 0,75 |
| P48025 | Tyrosine-protein kinase SYK | 3 | 0,0098 | 3 | 0,0105 | 2 | 0,0064 | 1 | 0,0035 | 0,0075 | 0,75 |
| Q8BVG5 | Polypeptide N-acetylgalactosaminyltransferase 14 | 3 | 0,0098 | 3 | 0,0105 | 2 | 0,0064 | 1 | 0,0035 | 0,0075 | 0,75 |
| Q61191 | Host cell factor 1 | 5 | 0,0163 | 2 | 0,0070 | 1 | 0,0032 | 1 | 0,0035 | 0,0075 | 0,75 |
| P15379 | CD44 antigen | 0 | 0,0000 | 4 | 0,0140 | 2 | 0,0064 | 2 | 0,0071 | 0,0069 | 0,69 |
| Q9QZM8 | F-box only protein 17 | 2 | 0,0065 | 2 | 0,0070 | 1 | 0,0032 | 3 | 0,0106 | 0,0068 | 0,68 |
| Q921L8 | Polypeptide N-acetylgalactosaminyltransferase 11 | 3 | 0,0098 | 1 | 0,0035 | 1 | 0,0032 | 3 | 0,0106 | 0,0068 | 0,68 |
| Q8BPP1 | Protein mab-21-like 2 | 1 | 0,0033 | 3 | 0,0105 | 3 | 0,0096 | 1 | 0,0035 | 0,0067 | 0,67 |
| Q80VA0 | N-acetylgalactosaminyltransferase 7 | 1 | 0,0033 | 4 | 0,0140 | 2 | 0,0064 | 1 | 0,0035 | 0,0068 | 0,68 |
| Q91V98 | Endosialin | 2 | 0,0065 | 4 | 0,0140 | 2 | 0,0064 | 0 | 0,0000 | 0,0067 | 0,67 |
| O08832 | Polypeptide N-acetylgalactosaminyltransferase 4 | 0 | 0,0000 | 1 | 0,0035 | 2 | 0,0064 | 4 | 0,0141 | 0,0060 | 0,60 |
| Q80ZA0 | Intelectin-1b | 0 | 0,0000 | 2 | 0,0070 | 2 | 0,0064 | 3 | 0,0106 | 0,0060 | 0,60 |
| Q8C7U7 | Polypeptide N-acetylgalactosaminyltransferase 6 | 1 | 0,0033 | 3 | 0,0105 | 0 | 0,0000 | 3 | 0,0106 | 0,0061 | 0,61 |
| Q6P9S7 | Polypeptide N-acetylgalactosaminyltransferase 10 | 1 | 0,0033 | 1 | 0,0035 | 3 | 0,0096 | 2 | 0,0071 | 0,0058 | 0,58 |
| Q07797 | Galectin-3-binding protein | 2 | 0,0065 | 1 | 0,0035 | 1 | 0,0032 | 3 | 0,0106 | 0,0060 | 0,60 |
| Q8VEH8 | Endoplasmic reticulum lectin 1 | 2 | 0,0065 | 1 | 0,0035 | 2 | 0,0064 | 2 | 0,0071 | 0,0059 | 0,59 |
| O88200 | C-type lectin domain family 11 member A | 2 | 0,0065 | 1 | 0,0035 | 3 | 0,0096 | 1 | 0,0035 | 0,0058 | 0,58 |
| P22366 | Myeloid differentiation primary response protein MyD88 | 2 | 0,0065 | 1 | 0,0035 | 3 | 0,0096 | 1 | 0,0035 | 0,0058 | 0,58 |
| O08859 | Tumor necrosis factor-inducible gene 6 protein | 1 | 0,0033 | 2 | 0,0070 | 4 | 0,0127 | 0 | 0,0000 | 0,0057 | 0,57 |
| Q7TQD2 | Tubulin polymerization-promoting protein | 3 | 0,0098 | 1 | 0,0035 | 1 | 0,0032 | 2 | 0,0071 | 0,0059 | 0,59 |
| O70340 | Neuronal pentraxin-2 | 2 | 0,0065 | 3 | 0,0105 | 1 | 0,0032 | 1 | 0,0035 | 0,0059 | 0,59 |
| Q62230 | Sialoadhesin | 1 | 0,0033 | 5 | 0,0175 | 1 | 0,0032 | 0 | 0,0000 | 0,0060 | 0,60 |
| P42227 | Signal transducer and activator of transcription 3 | 4 | 0,0131 | 0 | 0,0000 | 1 | 0,0032 | 2 | 0,0071 | 0,0058 | 0,58 |
| Q61282 | Aggrecan core protein | 3 | 0,0098 | 2 | 0,0070 | 2 | 0,0064 | 0 | 0,0000 | 0,0058 | 0,58 |
| Q9D0F3 | Protein ERGIC-53 | 0 | 0,0000 | 1 | 0,0035 | 2 | 0,0064 | 3 | 0,0106 | 0,0051 | 0,51 |
| P06745 | Glucose-6-phosphate isomerase | 0 | 0,0000 | 3 | 0,0105 | 1 | 0,0032 | 2 | 0,0071 | 0,0052 | 0,52 |
| P20917 | Myelin-associated glycoprotein | 2 | 0,0065 | 1 | 0,0035 | 0 | 0,0000 | 3 | 0,0106 | 0,0052 | 0,52 |
| P08551 | Neurofilament light polypeptide | 1 | 0,0033 | 2 | 0,0070 | 1 | 0,0032 | 2 | 0,0071 | 0,0051 | 0,51 |
| Q9JI58 | Retinoic acid early-inducible protein 1-delta | 1 | 0,0033 | 2 | 0,0070 | 1 | 0,0032 | 2 | 0,0071 | 0,0051 | 0,51 |
| P39061 | Collagen alpha-1(XVIII) chain | 2 | 0,0065 | 0 | 0,0000 | 2 | 0,0064 | 2 | 0,0071 | 0,0050 | 0,50 |
| Q8BGT9 | Polypeptide N-acetylgalactosaminyltransferase 12 | 2 | 0,0065 | 0 | 0,0000 | 2 | 0,0064 | 2 | 0,0071 | 0,0050 | 0,50 |
| Q8C1T8 | C-type lectin domain family 2 member H | 1 | 0,0033 | 1 | 0,0035 | 3 | 0,0096 | 1 | 0,0035 | 0,0050 | 0,50 |
| P21855 | B-cell differentiation antigen CD72 | 2 | 0,0065 | 1 | 0,0035 | 1 | 0,0032 | 2 | 0,0071 | 0,0051 | 0,51 |
| Q7TT15 | Polypeptide N-acetylgalactosaminyltransferase 17 | 2 | 0,0065 | 1 | 0,0035 | 2 | 0,0064 | 1 | 0,0035 | 0,0050 | 0,50 |
| Q60660 | Killer cell lectin-like receptor 2 | 2 | 0,0065 | 1 | 0,0035 | 3 | 0,0096 | 0 | 0,0000 | 0,0049 | 0,49 |
| Q9D9Q6 | Calreticulin-3 | 2 | 0,0065 | 2 | 0,0070 | 2 | 0,0064 | 0 | 0,0000 | 0,0050 | 0,50 |
| Q8VCD3 | Protein ERGIC-53-like | 2 | 0,0065 | 3 | 0,0105 | 1 | 0,0032 | 0 | 0,0000 | 0,0051 | 0,51 |
| Q8CJC7 | Killer cell lectin-like receptor subfamily E member 1 | 0 | 0,0000 | 2 | 0,0070 | 1 | 0,0032 | 2 | 0,0071 | 0,0043 | 0,43 |
| P31809 | Carcinoembryonic antigen-related cell adhesion molecule 1 | 1 | 0,0033 | 0 | 0,0000 | 2 | 0,0064 | 2 | 0,0071 | 0,0042 | 0,42 |
| Q08879 | Fibulin-1 | 1 | 0,0033 | 0 | 0,0000 | 2 | 0,0064 | 2 | 0,0071 | 0,0042 | 0,42 |
| Q80W49 | Beta/gamma crystallin domain-containing protein 3 | 1 | 0,0033 | 1 | 0,0035 | 1 | 0,0032 | 2 | 0,0071 | 0,0043 | 0,43 |
| Q91ZW7 | CD209 antigen-like protein E | 1 | 0,0033 | 2 | 0,0070 | 0 | 0,0000 | 2 | 0,0071 | 0,0043 | 0,43 |
| A2AKB9 | DDB1- and CUL4-associated factor 10 | 1 | 0,0033 | 1 | 0,0035 | 2 | 0,0064 | 1 | 0,0035 | 0,0042 | 0,42 |
| Q60653 | Killer cell lectin-like receptor 6 | 1 | 0,0033 | 1 | 0,0035 | 2 | 0,0064 | 1 | 0,0035 | 0,0042 | 0,42 |
| Q8K1B9 | Polypeptide N-acetylgalactosaminyltransferase 18 | 2 | 0,0065 | 0 | 0,0000 | 2 | 0,0064 | 1 | 0,0035 | 0,0041 | 0,41 |
| Q9JL99 | C-type lectin domain family 1 member B | 1 | 0,0033 | 1 | 0,0035 | 3 | 0,0096 | 0 | 0,0000 | 0,0041 | 0,41 |
| O54707 | Natural killer cells antigen CD94 | 2 | 0,0065 | 1 | 0,0035 | 1 | 0,0032 | 1 | 0,0035 | 0,0042 | 0,42 |
| P20693 | Low affinity immunoglobulin epsilon Fc receptor | 2 | 0,0065 | 1 | 0,0035 | 1 | 0,0032 | 1 | 0,0035 | 0,0042 | 0,42 |
| P37217 | Early activation antigen CD69 | 2 | 0,0065 | 1 | 0,0035 | 1 | 0,0032 | 1 | 0,0035 | 0,0042 | 0,42 |
| Q60652 | Killer cell lectin-like receptor 5 | 1 | 0,0033 | 2 | 0,0070 | 2 | 0,0064 | 0 | 0,0000 | 0,0042 | 0,42 |
| O08912 | Polypeptide N-acetylgalactosaminyltransferase 1 | 2 | 0,0065 | 2 | 0,0070 | 0 | 0,0000 | 1 | 0,0035 | 0,0043 | 0,43 |
| P15306 | Thrombomodulin | 1 | 0,0033 | 3 | 0,0105 | 1 | 0,0032 | 0 | 0,0000 | 0,0042 | 0,42 |
| Q7TSQ1 | C-type lectin domain family 18 member A | 2 | 0,0065 | 1 | 0,0035 | 2 | 0,0064 | 0 | 0,0000 | 0,0041 | 0,41 |
| P43025 | Tetranectin | 0 | 0,0000 | 1 | 0,0035 | 1 | 0,0032 | 2 | 0,0071 | 0,0034 | 0,34 |
| Q8BRU4 | C-type lectin domain family 9 member A | 0 | 0,0000 | 1 | 0,0035 | 1 | 0,0032 | 2 | 0,0071 | 0,0034 | 0,34 |
| Q9JJ61 | Polypeptide N-acetylgalactosaminyltransferase 16 | 1 | 0,0033 | 0 | 0,0000 | 1 | 0,0032 | 2 | 0,0071 | 0,0034 | 0,34 |
| Q8BHC0 | Lymphatic vessel endothelial hyaluronic acid receptor 1 | 0 | 0,0000 | 1 | 0,0035 | 2 | 0,0064 | 1 | 0,0035 | 0,0033 | 0,33 |
| Q8VCK6 | Free fatty acid receptor 2 | 1 | 0,0033 | 1 | 0,0035 | 0 | 0,0000 | 2 | 0,0071 | 0,0035 | 0,35 |
| Q8VED9 | Galectin-related protein | 1 | 0,0033 | 1 | 0,0035 | 0 | 0,0000 | 2 | 0,0071 | 0,0035 | 0,35 |
| P43137 | Lithostathine-1 | 0 | 0,0000 | 2 | 0,0070 | 1 | 0,0032 | 1 | 0,0035 | 0,0034 | 0,34 |
| P15501 | Prostatic spermine-binding protein | 1 | 0,0033 | 0 | 0,0000 | 2 | 0,0064 | 1 | 0,0035 | 0,0033 | 0,33 |
| P98064 | Mannan-binding lectin serine protease 1 | 1 | 0,0033 | 0 | 0,0000 | 2 | 0,0064 | 1 | 0,0035 | 0,0033 | 0,33 |
| Q6DIA9 | F-box only protein 27 | 2 | 0,0065 | 1 | 0,0035 | 0 | 0,0000 | 1 | 0,0035 | 0,0034 | 0,34 |
| Q80ZE3 | Sialic acid-binding Ig-like lectin 10 | 2 | 0,0065 | 1 | 0,0035 | 0 | 0,0000 | 1 | 0,0035 | 0,0034 | 0,34 |
| Q8K0C5 | Zymogen granule membrane protein 16 | 2 | 0,0065 | 1 | 0,0035 | 0 | 0,0000 | 1 | 0,0035 | 0,0034 | 0,34 |
| Q9EPW4 | C-type lectin domain family 3 member A | 2 | 0,0065 | 1 | 0,0035 | 1 | 0,0032 | 0 | 0,0000 | 0,0033 | 0,33 |
| P24721 | Asialoglycoprotein receptor 2 | 0 | 0,0000 | 1 | 0,0035 | 1 | 0,0032 | 1 | 0,0035 | 0,0026 | 0,26 |
| Q8BNX1 | C-type lectin domain family 4 member G | 0 | 0,0000 | 1 | 0,0035 | 1 | 0,0032 | 1 | 0,0035 | 0,0026 | 0,26 |
| P58659 | Protein eva-1 homolog C | 1 | 0,0033 | 0 | 0,0000 | 1 | 0,0032 | 1 | 0,0035 | 0,0025 | 0,25 |
| Q9D1H9 | Microfibril-associated glycoprotein 4 | 1 | 0,0033 | 0 | 0,0000 | 1 | 0,0032 | 1 | 0,0035 | 0,0025 | 0,25 |
| O54709 | NKG2-D type II integral membrane protein | 1 | 0,0033 | 1 | 0,0035 | 0 | 0,0000 | 1 | 0,0035 | 0,0026 | 0,26 |
| P97290 | Plasma protease C1 inhibitor | 1 | 0,0033 | 1 | 0,0035 | 0 | 0,0000 | 1 | 0,0035 | 0,0026 | 0,26 |
| Q61361 | Brevican core protein | 1 | 0,0033 | 1 | 0,0035 | 0 | 0,0000 | 1 | 0,0035 | 0,0026 | 0,26 |
| O09049 | Regenerating islet-derived protein 3-gamma | 1 | 0,0033 | 1 | 0,0035 | 1 | 0,0032 | 0 | 0,0000 | 0,0025 | 0,25 |
| Q80WM4 | Hyaluronan and proteoglycan link protein 4 | 1 | 0,0033 | 1 | 0,0035 | 1 | 0,0032 | 0 | 0,0000 | 0,0025 | 0,25 |
|  | Total | 306 |  | 286 |  | 314 |  | 283 |  | 1 | 100 |
